# Towards Decrypting Cryptobiosis—Analyzing Anhydrobiosis in the Tardigrade *Milnesium tardigradum* Using Transcriptome Sequencing

**DOI:** 10.1371/journal.pone.0092663

**Published:** 2014-03-20

**Authors:** Chong Wang, Markus A. Grohme, Brahim Mali, Ralph O. Schill, Marcus Frohme

**Affiliations:** 1 Molecular Biotechnology and Functional Genomics, Technical University of Applied Sciences Wildau, Wildau, Germany; 2 Biological Institute, Zoology, University of Stuttgart, Stuttgart, Germany; University of North Carolina at Charlotte, United States of America

## Abstract

**Background:**

Many tardigrade species are capable of anhydrobiosis; however, mechanisms underlying their extreme desiccation resistance remain elusive. This study attempts to quantify the anhydrobiotic transcriptome of the limno-terrestrial tardigrade *Milnesium tardigradum*.

**Results:**

A prerequisite for differential gene expression analysis was the generation of a reference hybrid transcriptome atlas by assembly of Sanger, 454 and Illumina sequence data. The final assembly yielded 79,064 contigs (>100 bp) after removal of ribosomal RNAs. Around 50% of them could be annotated by SwissProt and NCBI non-redundant protein sequences. Analysis using CEGMA predicted 232 (93.5%) out of the 248 highly conserved eukaryotic genes in the assembly. We used this reference transcriptome for mapping and quantifying the expression of transcripts regulated under anhdydrobiosis in a time-series during dehydration and rehydration. 834 of the transcripts were found to be differentially expressed in a single stage (dehydration/inactive tun/rehydration) and 184 were overlapping in two stages while 74 were differentially expressed in all three stages. We have found interesting patterns of differentially expressed transcripts that are in concordance with a common hypothesis of metabolic shutdown during anhydrobiosis. This included down-regulation of several proteins of the DNA replication and translational machinery and protein degradation. Among others, heat shock proteins Hsp27 and Hsp30c were up-regulated in response to dehydration and rehydration. In addition, we observed up-regulation of ployubiquitin-B upon rehydration together with a higher expression level of several DNA repair proteins during rehydration than in the dehydration stage.

**Conclusions:**

Most of the transcripts identified to be differentially expressed had distinct cellular function. Our data suggest a concerted molecular adaptation in *M*. *tardigradum* that permits extreme forms of ametabolic states such as anhydrobiosis. It is temping to surmise that the desiccation tolerance of tradigrades can be achieved by a constitutive cellular protection system, probably in conjunction with other mechanisms such as rehydration-induced cellular repair.

## Introduction

Tardigrades or “water bears” are tiny aquatic or semi-aquatic invertebrates and comprise two main classes: Heterotardigrada and Eutardigrada [1]. Recent molecular studies [2], [3] have indicated that tardigrades have a sister group relationship with Onychophora and Arthropoda. Tardigrades can survive extreme environmental conditions by reversibly suspending their metabolism, a phenomenon known as cryptobiosis. Many tardigrade species are capable of anhydrobiosis, a form of cryptobiosis induced by desiccation [4]. Similar to other metazoans such as nematodes and rotifers, tardigrades are capable of anhydrobiosis at any stage of their life-cycle [1], [2]. Responding to desiccation, tardigrades form a so-called ‘tun’, observed as a constricted and folded body, and enter the state of anhydrobiosis [2]. The anhydrobiotic “tun stage” is highly resistant to abiotic factors, e.g. high temperature, high hydrostatic pressure and even high doses of radiation [5]–[7]. The study of tardigrade anhydrobiosis will help gain insight into dry preservation techniques for biological materials.

Although there has been progress in unravelling anhydrobiosis in tardigrades, most of the underlying mechanisms regarding their extreme desiccation resistance remain elusive [8], [9]. Desiccation in general causes severe damage of cellular structures, leading to the death of cells and the organism. Non-reducing disaccharides such as trehalose (e.g. in bacteria, fungi and animals) [1], [10] and sucrose (e.g. in desiccation-tolerant plant seeds) [11], [12] have been found to play a vital role in avoiding such a damage. Trehalose and sucrose protect cells and bio-molecules by (1) replacing water that is normally bonded to hydrogen, (2) participating in the formation of a glassy matrix in the cytoplasm or (3) helping in stabilization of dried DNA [11]–[14]. Nonetheless, there have been findings in some desiccation-tolerant species which do not support a universal protective role of the disaccharides in anhydrobiosis. For instance, bdelloid rotifers do not seem to accumulate trehalose [1], [14]. In all the species of Heterotardigrada investigated so far, no significant change in the trehalose level has been observed during anhydrobiotic states [15]. The *presence of the disaccharide trehalose in Milnesium tardigradum* has been a subject of debate. Horikawa et al. [16] were able to detect low amounts of trehalose in the anhydrobiotic *M. tardigradum* from a Japanese population. In contrast, Hengherr et al. [15] reported *M. tardigradum* as lacking treholose, although they identified trehalose in other seven investigated tardigrade species. A recent study by Jönsson and Persso [17] has provided further evidence on the presence of very low amounts of trehalose in *M. tardigradum* from a Swedish population, but no increase of trehalose was observed in connection with desiccation. The protection by non-reducing disaccharides does not seem to be a ubiquitous mechanism accounting for anhydrobiosis in all desiccation-tolerant organisms. This leads to the question as to whether other molecular adaptations are required for anhydrobiosis. To date, several members of stress protein families and hydrophilic proteins have been implicated in desiccation tolerance. Functioning as protein chaperones, heat shock proteins (Hsps) are believed to assist nascent and misfolded proteins to gain their correct conformation [15]. In contrast, hydrophilic late embryogenesis abundant (LEA) proteins are presumed to provide resistance to water stress conditions by avoiding aggregation [18]. The up-regulation of the genes encoding Hsps has been observed in diverse desiccation-tolerant organisms under drought or desiccation stress, e.g. the cyanobacterium *Anabaena* sp. PCC7120 [19], the desert-dwelling species S*phincterochila zonata* [20], the resurrection plant *Haberlea rhodopensis* [21], or the sleeping chironomid *Polypedilum vanderplanki*, the largest anhydrobiotic animal known up to now [22]. Previous studies have yielded unequivocal findings about the transcriptional response of Hsps to desiccation in tardigrades. For instance, Jönsson and Schill [23] have detected a down-regulation of Hsp70 in desiccated specimens of eutardigrade *Richtersius coronifer*, whereas Altiero et al. [24] reported that the expression levels of Hsp70 are similar between dehydrated and active specimens of eutardigrade *Bertolanius volubilis*. In eutardigrade *M. tardigradum*, one isoform of the Hsp70 family gene shows an up-regulation during the induction of the desiccated state, while the other two isoforms have significantly lower levels of mRNA expression in the dehydated state than in the hydrated state [25]. In the *Bertolanius volubilis* investigated by Altiero et al. [24], the dehydration stress does not induce an increase in Hsp90 expression either. In contrast to this finding, Hsp90 in eutardigrade *M. tardigradum* has been found to be significantly up-regulated in the anhydrobiotic state [26]. Hydrophilic LEA proteins were first reported in resurrection plants and later in non-plant organisms [1]. For instance, in nematodes [27]–[29], rotifers [30] as well as in plants [21], the induction of LEA proteins has been associated with dehydration tolerance. The evaluation of LEA proteins in tardigrades is very recent and leads to some contradictory data too. The presence of LEA-like transcripts and protein was detected by a few studies of tardigrades using expressed sequence tag (EST) or proteomics [31]–[33]. LEA proteins are heat-soluble molecules considering their biochemical property. Recently, a heat-soluble proteomics study has showed that abundant heat-soluble proteins bear no sequence similarity with LEA proteins and stress-induced novel protein families have distinct sub-cellular localizations in an anhydrobiotic tardigrade *Ramazzottius varieornatus* [34].

Advances in the field of high-throughput sequencing, RNA sequencing (RNA-Seq) in particular, have revolutionized transcriptomic studies and enabled researchers to study transcriptomes at unprecedented detail [35]. In recent years, there has been an increasing interest in mechanistic analyses of anhydrobiosis using next-generation sequencing (NGS). For instance, transcriptome analysis by NGS [21] has revealed a drought-induced reprogramming in glacial relic *Haberlea rhodopensis*, a resurrection plant with remarkable tolerance to desiccation. The reprogramming redirects resources from growth towards cell protection [21]. Molecular analysis using ESTs has revealed the transcripts differentially expressed in an Antarctic nematode *Plectus murrayi* during desiccation stress [29], thereby several desiccation-induced transcriptomes have been identified, including trehalose, ribosomal proteins and transcripts similar to plant LEA related family members of *Caenorhabditis elegans*. In our previous comparative analysis of *M. tardigradum* and *Hypsibius dujardini*, we found several clearly over-represented motifs important for mRNA storage and stability such as lox P DICE elements implicated in tardigrade stress adaptations [33], [36].

This paper seeks to address the transcriptional response to desiccation in the tardigrade *M. tardigradum*. Four different stages were investigated in this study, including active, dehydration, inactive tun and rehydration stages. To generate a comprehensive transcriptome assembly, we combined previously generated cDNA clone data [36] and high-throughput sequencing datasets from 454 and Illumina sequencing. Hybrid transcriptome sequencing and assembly is still a largely unsolved problem where no best practices exist due to the ongoing evolution of the sequencing chemistries and an ever growing number of sequencing technologies [37], [38]. We therefore employed a custom pipeline of publicly available tools that fit our available sequencing data. This involved error correction and pre-assembly of the short read datasets using programs specialized for the respective data types. As no genomic data was available, *de novo* assembly was performed.

## Results and Discussion

### *De novo* transcriptome assembly

**Figure 1:**
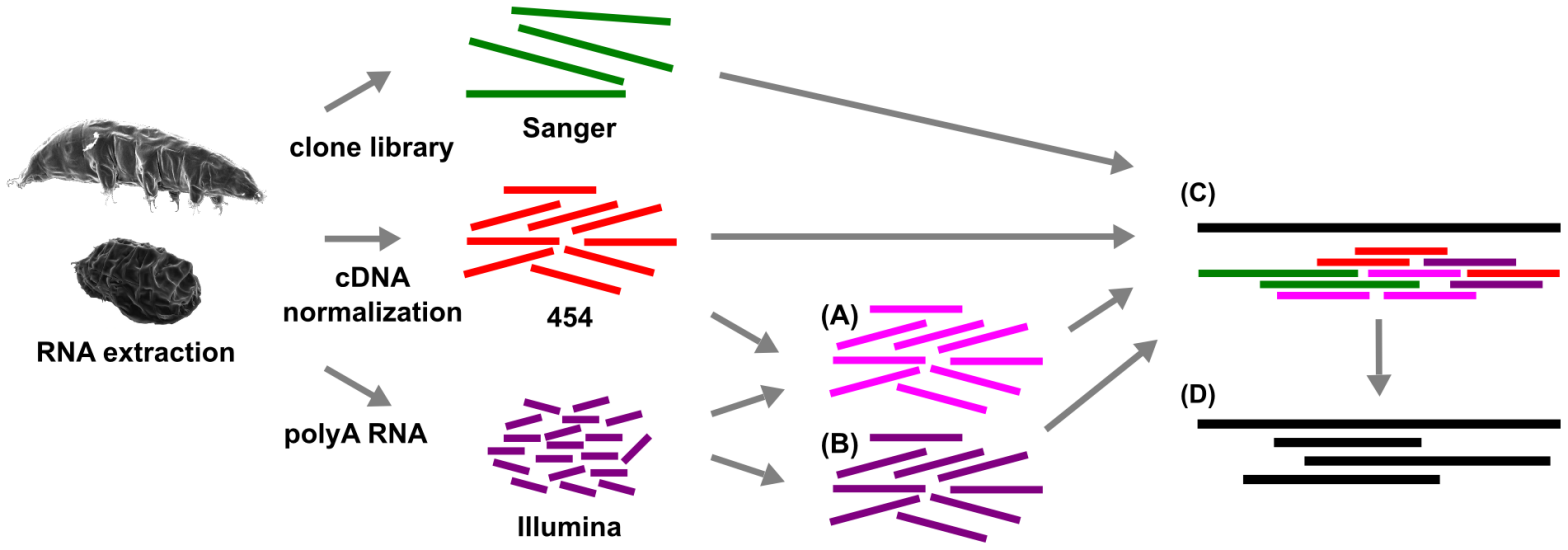
Workflow for the de novo hybrid assembly of the *M. tardigradum* transcriptome. (A) Hybrid assembly of 454 and Illumina using ABySS (AbySS_hyb). (B) Illumina assembly using (AbySS_sol). (C) Hybrid assembly of Sanger, 454 and ABySS contigs using MIRA. (D) Final clustering and assembly using TGICL.

For performing hybrid *de novo* transcriptome assembly, we chose to combine “de Bruijn graph (DBG)” [39], [40] and “overlap layout consensus (OLC)” [41] assemblers to make use of their advantages (Figure 1). We generated longer contigs from short high-throughput sequencing data using a DBG assembler, thereby greatly reducing the amount of data that had to be assembled. We then assembled the original longer reads together with the resulting contigs as pseudo-reads using an OLC assembler, thereby guiding the assembly with the provided pre-assembled contigs.

**Figure 2:**
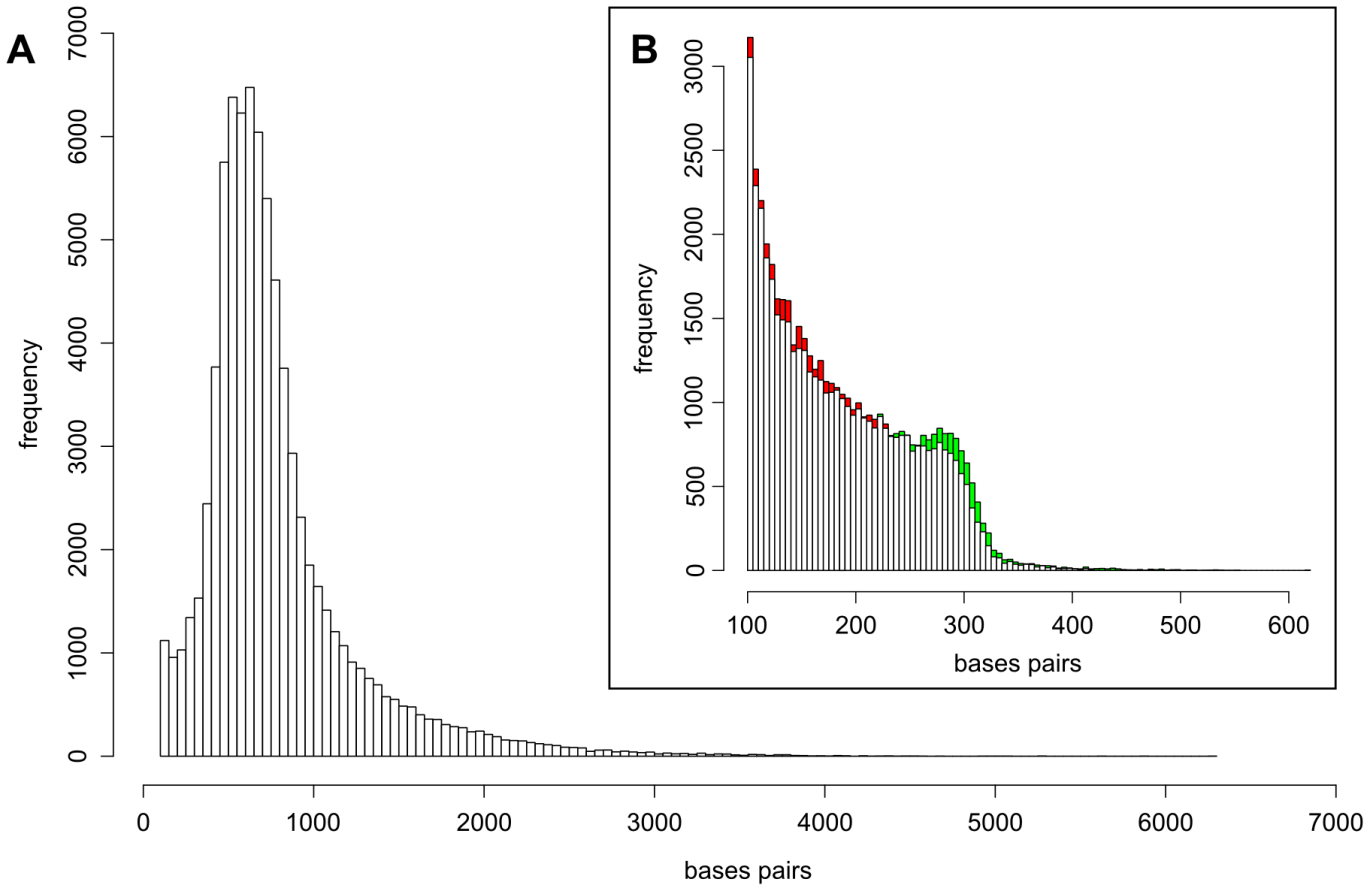
Transcriptome assembly contig distributions. (A) Final assembly contig length distribution. (B) Illumina data assembly contig length distribution before and after error correction using Reptile (red: decrease, green: increase).

Datasets used for transcriptome assembly were ∼1.18 Mio 454 GS FLX Titanium reads (392.03 Mb) and ∼61.6 Mio Illumina reads from four lanes on an Illumina GAII (2217.63 Mb). Due to the short nature of 454 sequencing (mean length 331 bases) and Illumina sequencing (36 bases, single end sequencing), the NGS data were pre-assembled (see procedures in Figure 1). This was done using a DBG assembler optimized for short read data namely ABySS [42] as the vast amount of sequence data typically cannot be handled by assemblers that are optimized for Sanger and low coverage 454 data. The Illumina dataset was error-corrected using Reptile [43]. The 454 datasets were pooled with the Reptile corrected Illumina reads and errors were then corrected using hybrid-SHREC [44]. The clustering tool CD-HIT-EST [45] was used to generate a non-redundant dataset that contains only unique contigs from all ABySS assemblies. The final merged Illumina dataset (ABySS_sol) contained 48,483 contigs with at least 100 bp. The amount of data was ∼9.13 Mb with a mean read length of 189 bp and a median of 177 bp (Figure 1B). The 41,536 hybrid 454+ Illumina non-redundant contigs (ABySS_hyb) of at least 100 bp contained ∼20.65 Mb of data, with a mean of 497 bp and a median of 298 bp. Subsequently, the Sanger, 454 reads plus the two pre-assembled datasets were combined in a single assembly using the OLC assembler MIRA [41]. As MIRA is rather conservative and some contigs were not assembled due to local homopolymer errors (procedure in Figure 1D), the sequences were clustered and assembled in a final step using TGICL [46]. Unlike MIRA, the TGICL pipeline is not capable of masking input data on its own. Therefore, the contigs were softmasked (lower case) using RBR [47] prior to clustering and assembly using TGICL. The final assembly contig distribution is shown in Figure 2A. The impact on error correction on the assembly of Illumina data (ABySS_sol) shows an increase of the fraction of longer contigs peaking at ∼300 bp, along with a decrease in the abundance of smaller contigs (Figure 2B). This hints at better assembly contiguity, where previously unassembled fragments were joined into longer contigs. The distinct peak at 300 bp marks the physical fragmentation range of the input DNA for library preparation. The maximum contig length is probably slightly shorter than the original cDNA molecules as polyA tails longer than the original read length cannot be reconstructed. Scaffolding yielded 52 scaffolds by mapping the translation of the remaining contigs on the reference *Drosophila melanogaster* proteome. Of these scaffolds, 14 could be assembled directly into a single contiguous stretch, 31 contained a single gap and three scaffolds exhibited two gaps. The largest gap had 4922 bases and the shortest had six bases. The scaffolding of the remaining contigs did generate rather few scaffolds. This can have two reasons: the remaining reads and contigs introduced into the scaffolding pipeline were of low quality and could not be utilized; or there were not many possibilities left for scaffolding the residual data as the assembly was already saturated.

### Quality assessment of transcriptome assembly

Contig length might be a good estimator for assembly quality in genome sequencing projects, but has probably little informative value in transcriptome assemblies as average contig length is correlated with original transcript length and read coverage. In the current study, we evaluated the quality of the resulting assembly in two ways: using core eukaryotic genes mapping approach (CEGMA) [48] and by comparison with publicly available sequence data for a set of species. The reference datasets (genome and predicted proteome sequences) used in this study were from *Anopheles gambiae, C. elegans, Daphnia pulex, D. melanogaster, Ixodes scapularis, Pristionchus pacificus*, *Strigamia maritima* and *Tetranychus urticae*, and retrieved from the ENSEMBL Metazoa website [49] (release 20).

CEGMA searches for a reference set of 458 core eukaryotic genes (CEGs) that occur in a wide range of eukaryotes, and reports the degree to which the gene set of a genome covers the CEGMA reference set. We used CEGMA to measure how completely the core eukaryotic genome is covered by the *M. tardigradum* transcriptome. CEGMA can distinguish between predicted genes in partial and in full-length (referred to as ‘complete’). Analysis of our transcriptome assembly using CEGMA software identified 205 (95%) out of the subset of 248 highly-conserved CEGs as ‘complete’ (>70% alignment length with core proteins) and 230 out of the 248 CEGs as ‘partial’ (Table 1). While 82% and 90.8%, respectively, out of the highly conserved groups 3 and 4 of CEGs (as defined in [50]) were identified, 72.7% and 85.7%, respectively, of the more divergent groups 1 and 2 of CEGs were detected (Table S1 in File S5). We observed an elevated proportion of partial genes compared with the complete ones for all the CEG groups. The proportion difference between the partial and full-length genes indicates that some genes were split over contigs. Our Illumina dataset was sampled mostly from the 3′ end of transcripts, thereby contributing lesser to the complete transcript reconstruction, and likely resulting in more partial transcripts detected. However, we noted that the difference became smaller with increased degree of conservation. There were less predicted CEGs split over contigs for group 4 than those genes in group 1. Hence, the *M. tardigradum* transcriptome exhibited a higher continuity for highly conserved groups than the predicted genes out of the more divergent groups.

**Table 1:** Comparison of *M. tardigradum* assembly with reference genomes for the presence of 248 CEGs using CEGMA.

Organisms	# of 248 CEGs		*%* Completeness	
	Complete	Partial	Complete	Partial
*M. tardigrade*	205	232	82.66	93.55
*An. gambiae*	238	244	95.97	98.39
*C.elegans*	221	227	89.11	91.53
*D. pulex*	239	242	96.37	97.58
*D. melanogaster*	241	245	97.18	98.79
*I. scapularis*	105	199	42.34	80.24
*P. pacificus*	210	231	84.68	93.15
*S. maritima*	241	244	97.18	98.39
*T. urticae*	236	243	95.16	97.98

(“# of 248 CEGs”- 248 ultra-conserved core eukaryotic genes; “*%* Completeness”- percentage of 248 CEGs present).

For comparison we assessed the publicly available and well-assembled genomes of the above mentioned organisms. Using CEGMA, we determined the presence of 42–97% complete and 80–98% partial CEGs in those reference genomes (Table 1 and Table S1 in File S5). The *M. tardigradum* transcriptome shows a comparable continuity and a high detection level. In addition, CEGMA predicted an average of ∼2.45 orthologues per CEG, with 73.17% of detected CEGs having more than 1 orthologue for the *M. tardigradum* transcriptome. Repeating the analysis on the genome datasets of reference species detected an average of 1.20–1.43 orthologues per core protein. Our data are in line with those found in the *Nicotiana benthamiana* transcriptome [51]. An average of about 3 to 4 orthologues per core gene has been detected by Nakasugi et al. [52] based on their *Nicotiana benthamiana* transcriptome assembly and unigene transcriptome datasets. By comparison, the CEGMA assessment of the *Arabidopsis thaliana* genome shows on average 2 orthologues per core gene [52]. The orthologues prediction based on the transcriptome data tended to be higher than the genomic assessment. A little bit higher orthologue number per transcript is likely the impacted by the inherent redundancy of transcriptome assemblies.

**Figure 3:**
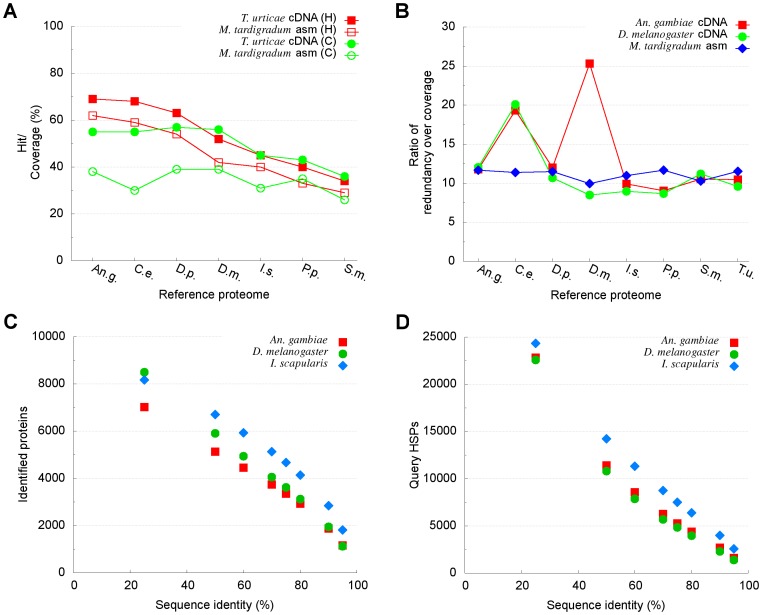
Representative results of proteome coverage analysis using est2assmbly. Reference cDNA and proteome data were from *Anopheles gambiae* (*An.g.*), *Caenorhabditis elegans* (*C.e.*), *Daphnia pulex* (*D.p.*), *Drosophila melanogaster* (*D.m.*), *Ixodes scapularis* (*I.s.*), *Pristionchus pacificus* (*P.p*.), *Strigamia Maritima* (*S.m*.) and *Tetranychus urticae* (*T.u*.). (A) Unique hits on reference proteomes aligned to the *M. tardigradum* transcriptome (open square) and *T.u*. cDNA (solid square), respectively; and the reference proteome coverages when aligned to the *M. tardigradum* transcriptome (open circle) and *T.u*. CDNA (solid circle). (B) Ratio of redundancy over coverage for reference proteomes aligned to *An.g*. cDNA (solid square), *D.m*. cDNA (solid circle) and the *M. tardigradum* transcriptome (solid diamond). (C) Proteins identified in relation to sequence identity in *An.g*. (solid square), *D.m*. (solid circle) and *I.s*. (solid diamond) proteomes compared to *M. tardigradum*. (D) Distribution of query HSPs covered by *An.g*. (solid square), *D.m*. (solid circle) and *I.s*. (solid diamond) proteomes respectively.

Cross-dataset comparison was performed to estimate the proportion of the reference proteomes aligned to the assembly and vice versa to determine the proportion of the assembly aligning to the reference proteomes. Est2assembly [52] was run on the *M. tardigradum* transcriptome assembly against the proteome datasets of the above-mentioned reference organisms (e-value < =  1e−5; bit-score > = 50 bits). The proportion of unique hits in the reference proteomes was found to be the highest with *An. Gambi*ae (62%), and tended to decrease according to evolutionary distance (Table 2). There are similarities between the trend observed in this study with those described by Martínez-Barnetche et al. [53], who assessed the *Anopheles albimanus* transcriptome by BLASTx comparisons with a set of insect proteomes, including *An. Gambiae, D. melanogaster* and *I. Scapularis*. To further examine that this pattern is not likely due to just chance, we ran est2assembly on each of the reference cDNA sequences against the reference proteomes. We observed that the unique hits in the reference proteomes aligned to each cDNA sequence followed a similar pattern (Figure 3A). Provided that the number of unique hits represents the number of transcribed genes [54], reference proteome coverages may be interpreted as a complementary indication to gene finding. Est2assembly distinguishes between coverage and overlapping coverage (i.e. redundancy). The coverage is calculated on the basis of base pair by counting the unique hits to a specific base pair, whereas the overlapping coverage reports the total number of hits which a base pair (or amino acid; referred to as position in est2assembly) has received [52]. For instance, aligned to the *M. tardigradum* transcriptome, *An. Gambiae* was found to have 2,817,113 (38%) non-overlapping positions with a mean length of 339 and a median of 295, and 32,857,743 overlapping positions with a mean length of 3,961 and a median of 1,415. In contrast, *D. melanogaster* yielded 4,155,663 (35%) non-overlapping positions with a mean length of 389 and a median of 305, and 41,311,778 overlapping positions with a mean length of 3,869 and a median of 1,397.5. We noted that the reference proteome coverages were lower than those obtained with the reference proteomes aligned to individual reference cDNA sequences. The reason for this may be explained by several different factors: (1) uneven transcript sampling due to expression differences, (2) indel errors of 454 sequencing that mess up reading frames and therefore chop up BLAST hits into smaller pieces falling below threshold, and (3) sequence divergence/loss in tardigrades. Considering the considerably different sizes between the transcriptome assembly and the reference datasets, we examined the ratio of redundancy over coverage. The ratios for the reference proteomes aligned to the transcriptome assembly were in the range of 9.9 to 11.7 (Table 2). These values were comparable to those obtained with the reference proteomes aligned to the reference cDNA sequences except for *C. elegans* and *D. melanogaster* (Figure 3B). According to the distribution of protein identity estimated by est2assembly, 3,740 of the *An. gambiae* proteins could be aligned to the *M. tardigradum* assembly with at least 70% sequence identity, while 6,260 of query (assembly) high-scoring segment pairs were found to be covered by the *An. gambiae* proteome at the same percent sequence identity. By comparison, 4,054 of the *D. melanogaster* proteins were identified at greater than 70% identity, while 5,703 of query high-scoring segment pairs were covered by the reference *D. melanogaster* proteome with sequence identity no less than 70% (Figures 3C and 3D).

**Table 2:** Comparison of *M. tardigradum* assembly to reference proteomes.

Ref. Organism	Ref. DB Entries	Unique Hits (%)	Transcripts with Hit (%)	Ref. DB size (base)	Ref. DB Non-OLP (%)	Ratio of RoC
*An. gambiae*	13,368	62	41	7,323,844	38	11.664
*C.elegans*	22,868	40	37	10,041,318	31	11.364
*D. melanogaster*	17,851	59	41	11,839,160	35	9.941
*D. pulex*	29,790	33	43	9,854,871	30	11.473
*I. scapularis*	20,431	42	39	5,811,053	39	10.989
*P. pacificus*	29,195	29	33	8,456,047	26	11.656
*S. maritima*	14,936	54	43	8,456,047	39	10.284
*T. urticae*	17,914	44	39	6,479,589	38	11.521

(“DB” - database; “OLP” - overlapping position; “Roc” - redundancy over coverage).

We noted also that the proportion of non-overlapping positions was higher in the reference *I. scapularis* proteome than in the *An. Gambiae* and *D. melanogaster* proteomes (Table 2, Figures 3C and 3D). The *I. scapularis* dataset did not seem to follow the pattern that would be expected based on the degree of phylogenetic relationships. A possible explanation for this might be the considerably small size of the *I. scapularis* proteome dataset compared with the *M. tardigradum* transcriptome. Indeed, Martínez-Barnetche et al. [53] have also observed a higher coverage of Pediculus humanus than *D. melanogaster* with the *Anopheles albimanus* transcripts which is unanticipated according to evolutionary relationships. Among the reference proteomes they used, the *Pediculus humanus* dataset is the smallest one in size and also considerably smaller than the *Anopheles albimanus* transcriptome assembly. Other reasons for this are not clear but it may have something to do with methodological differences.

### Assembly annotation

**Figure 4:**
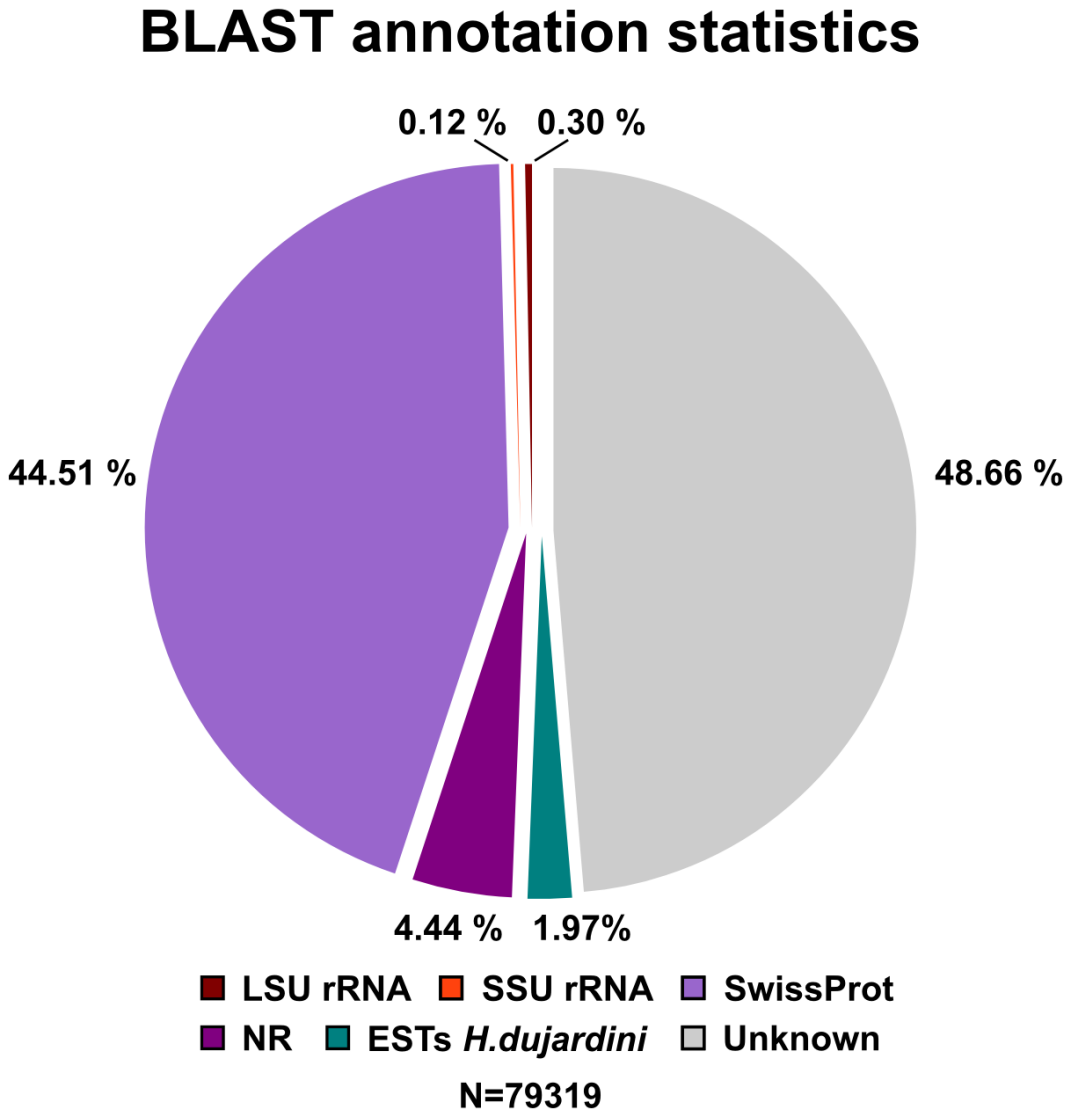
BLAST annotation of transcriptome assembly.

The final contigs were annotated using Blast2GO [55]. This was done in a successive fashion that the sequences were searched against databases of decreasing quality and only sequences without annotation were carried over to the next database search. The order of the databases were the SILVA database, containing small (16S/18S, SSU) and large subunit (23S/28S, LSU) ribosomal RNA (rRNA) sequences [56], Swiss-Prot [57], NCBI non-redundant protein database, and *H. dujardini* EST sequences (dbEST). Most of the sequences could be annotated using SwissProt, and NCBI non-redundant protein sequences (nr) added only a few additional annotations (Figure 3). The hits against the 5,235 *H. dujardini* ESTs available in dbEST seem to be tardigrade specific sequences. As shown in Figure 4, around 50% of the contigs could be annotated.

**Figure 5:**
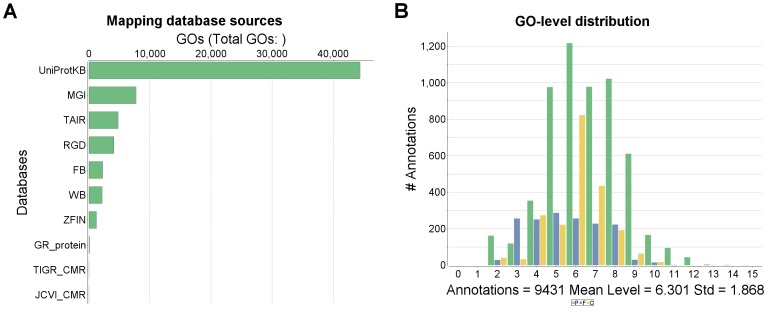
Annotation of CEGs identified in *M. tardigradum* transcriptome. (A) Databases from which annotations were retrieved. (B) Distribution of GO levels. P: protein, F: function, and C: component.

Analysis using CEGMA detected 408 of the set of 458 CEGs present in the *M. tardigradum* assembly (File S1). Of those predicted CEGs, 405 could be annotated using Swissprot. The database sources of mapping (Figure 5A) gives a detailed information on which sources annotations were retrieved using Blast2GO. Note that the GO level distribution of these predicted CEGs achieved a medium level of 6.031 with a standard deviation of 1.8 (Figure 5B). This average GO level is close to those documented in [55], thus suggesting a higher quality in the resulting annotation.

### Mapping reads to transcriptome and transcript quantification

**Figure 6:**
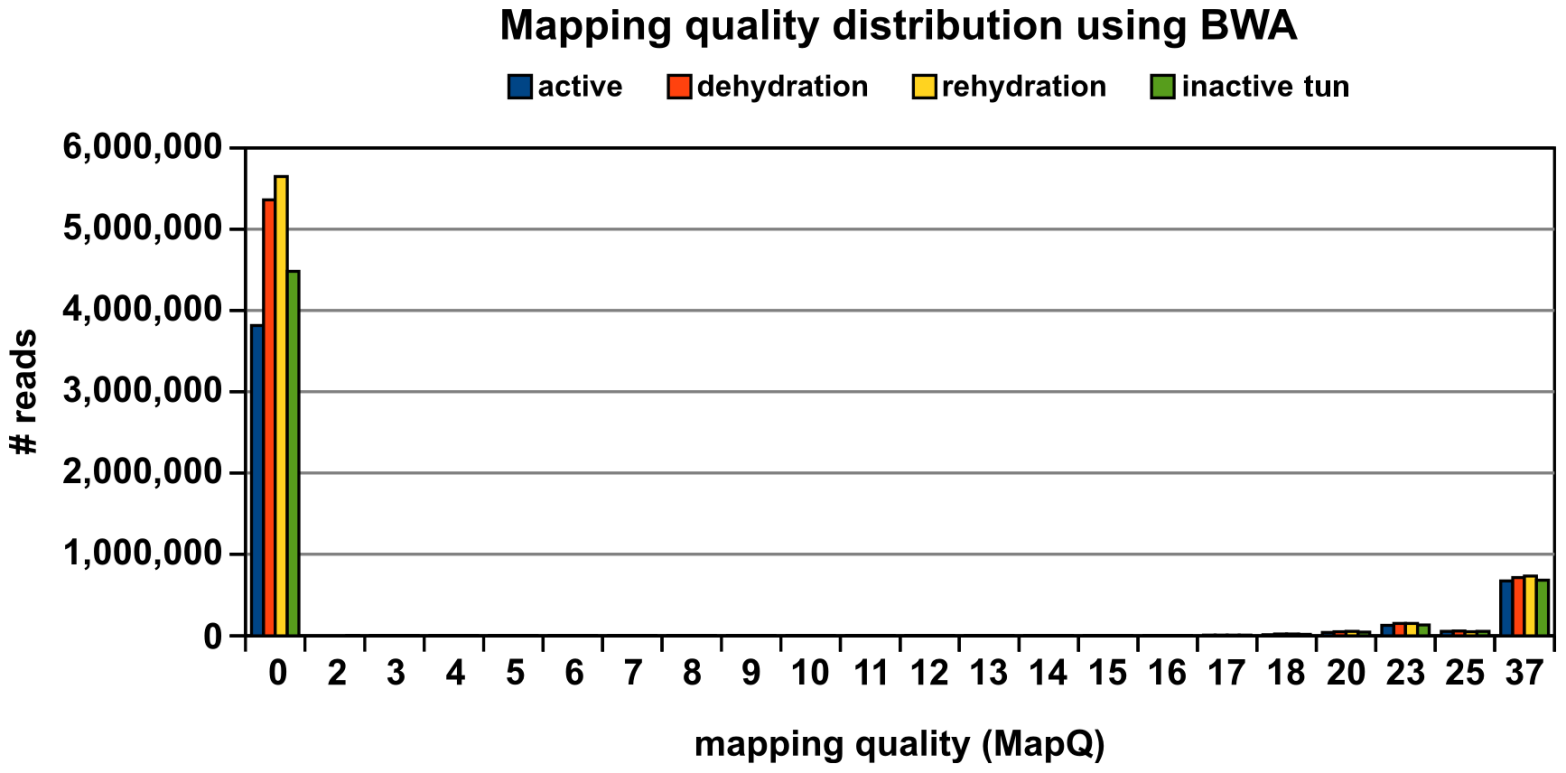
Mapping qualities of Illumina datasets mapped onto transcriptome assembly. MapQ 0: several equally best hits, MapQ 37: maximum MapQ for single end reads in BWA.

For mapping the Illumina reads onto the hybrid transcriptome assembly, a combination of two aligners was used, namely Burrows Wheeler Aligner (BWA) [58] and Stampy [59]. The mapping quality (MapQ) [60] was estimated using the formula: Q = −10log10 P, whereby the probability (P) measures how likely a read with a mapping quality (Q) is wrongly mapped. The mapping quality (MapQ) is hence the probability describing how likely a reported alignment represents the true alignment [57−>60]. For instance, a mapping quality of 30 means that there is a 1∶1000 chance that the alignment is wrong. If a read cannot be unambiguously mapped, most aligners assign a mapping quality of 0. In the current study, a majority of reads had a MapQ of 0 for all four Illumina datasets, as illustrated in Figure 6. On the other end of the spectrum, a minority of reads are shown with a MapQ of 37, suggesting perfect read matches. As the reads are derived from a window of 300–500 bp from the 3′ end of the transcripts, it is highly unlikely that the transcriptome of a simple organism such as *M. tardigradum* contains so many duplicated segments and the reads cannot be allocated due to this fact. For comparison, Li et al. [61] evaluated the uniqueness of reads on the mouse transcriptome by simulation studies, and found that 17% multi-reads are produced for 25 base reads. Increasing the length to 75 bases still result in 10% multi-reads, likely due to very similar isoforms. This bears no relation to the multi-read fraction found for *M. tardigradum* that ranges from 80–85% depending on the dataset. Therefore, one may speculate that the assembly reconstructed several isoforms that share most of their 3′ exons (in addition to redundant contigs and assembly artifacts).

**Figure 7:**
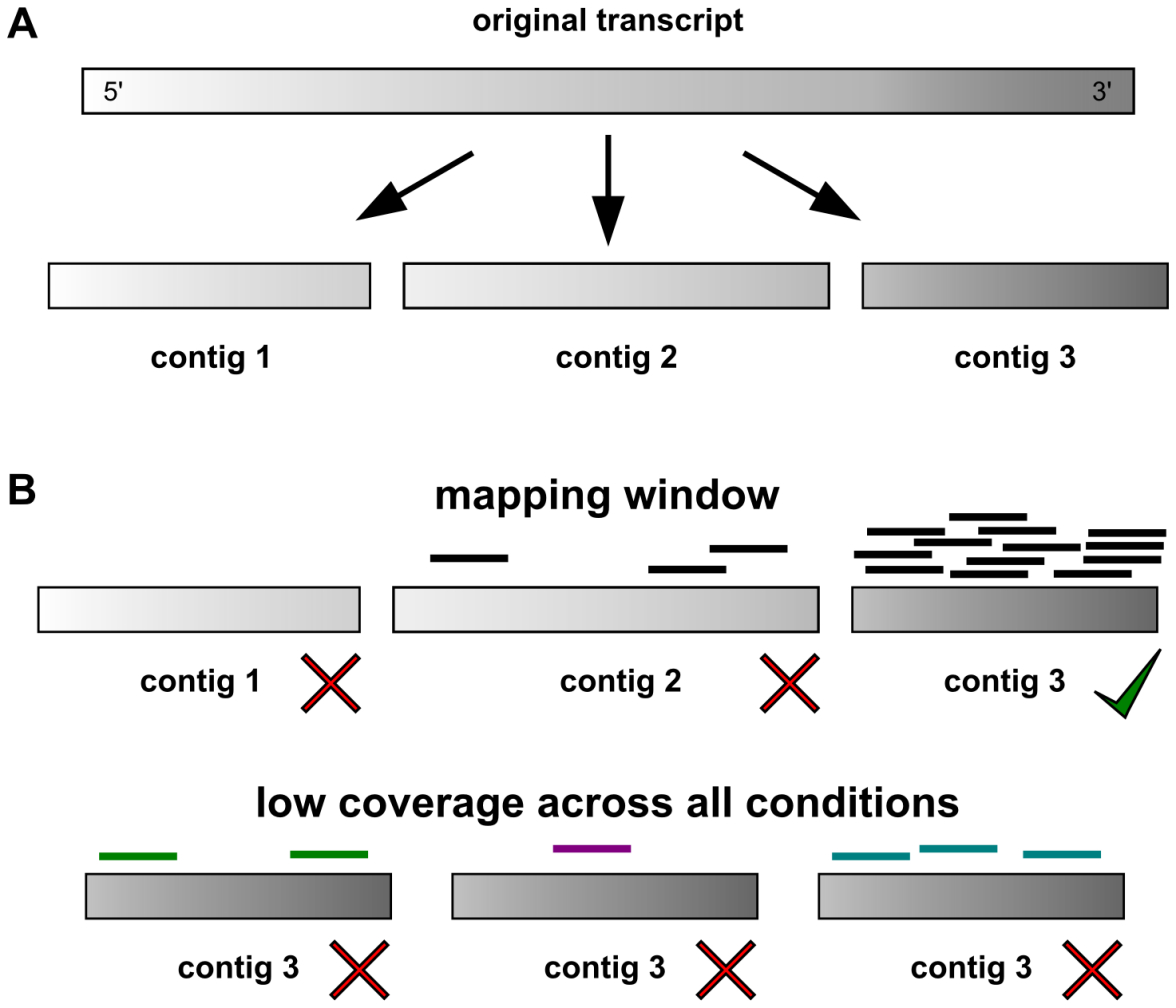
Filtering of reference transcripts for quantification. (A) Theoretical representation of original transcript as fragmented contigs in assembly. (B) Filtering of reference contigs by mapping window (top) or basal expression across all tested conditions (bottom).

The final assembly after the removal of ribosomal RNAs yielded 79,064 contigs larger than 100 bp that could not be collapsed further. Of these contigs, 6,994 did not have any Illumina read mapping in any of the stages and were removed. For quantification only the 3′ end of the reconstructed transcripts is important as this is the origin of the Illumina reads. The per transcript read count was summed over all samples and the lower 40% quantile was excluded from hypothesis testing in DESeq [62], resulting in the removal of all reference transcripts with a sum of 31 and less (normalized) reads mapped across all four samples. From the 72,070 reference contigs, a large fraction of 29,040 contigs (40.29%) was removed, including both basally expressed transcripts and also upstream parts of transcripts outside of the quantification window (Figure 7).

**Figure 8:**
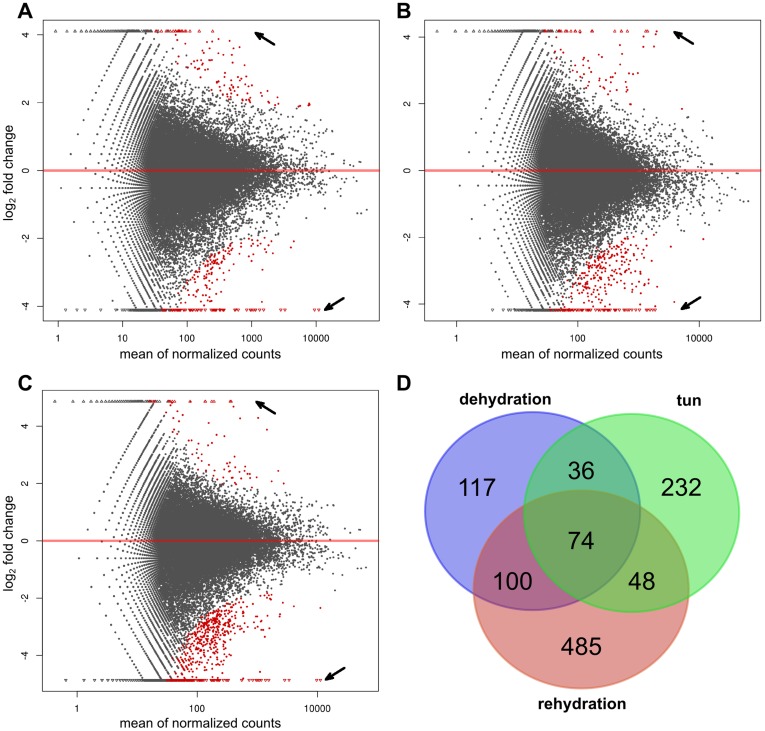
DESeq differential transcript expression analysis. (A–C) Log ratio versus abundance plots (MA-plot) for each of the anhydrobiotic stages versus active stage (A: active vs. dehydration; B: active vs. inactive tun; C: active vs rehydration; red dots: significantly regulated at FDR of 0.1, red triangle regions pointed by arrow: significantly regulated without counts in one of the stages and outliers). (D) Venn diagram of differentially expressed transcripts compared to active stage shared between stages (generated using http://bioinformatics.psb.ugent.be/webtools/Venn/).

By controlling the expected false discovery rate (FDR) to be no more than 0.1 [63], there were 327 significantly differentially expressed transcripts between active and dehydration animals (212 down/115 up), 390 between active and inactive tun (311 down/79 up) and 707 between active and rehydration animals (627 down/80 up) (Figures 8A–8D). The most striking result is that transcripts were mostly down-regulated in the three anhydrobiotic stages compared with the active stage. Unfortunately, more than 65% of the differentially expressed transcripts did not have any annotation assigned by BLAST analysis. The reason is probably a combination of tardigrade specific transcripts or that the contigs did not span coding sequence well enough to give significant BLAST hits. Some transcripts were found to be differentially expressed in more than one stage whereas 834 were only differentially expressed in a single stage. 184 were found to be differentially expressed in two of the stages and 74 could be found in all three stages (Figure 8D). Additionally, the complete expression count levels of the whole datasets of dehydration and rehydration were more similar to each other than to the inactive tun stage (not shown). The similar transcriptional response to dehydration and rehydration might in part due to a number of general stress-responsive genes as observed in the desiccation-tolerant *Anabaena* sp. PCC 7120 [64]. Such genes are supposed to be involved in switching from a maximal growth metabolism to a maintenance metabolism [65]. Another possible explanation for this is that some genes (e.g. like the stationary-phase-essential genes in *Saccharomyces cerevisiae* [66]) are not triggered by a specific environmental stress, but rather via reduced metabolic states. In spite of these speculations, the precise underlying molecular machinery remains to be determined.

### Differentially expressed transcripts

The following compilation is not exhaustive, but a summary of differentially expressed transcripts based on their well known function in the cell. Transcripts, which were found to be differentially expressed at all three anhydrobiotic stages and could be assigned to known genes, are shown in Table 3. Among the predicted CEGs, nine were found to be differentially expressed (Table S2 in File S5). A complete list of differentially expressed transcripts including their annotation can be found in Files S2, S3 and S4.

**Table 3:** Annotated differentially expressed transcripts in all three anhydrobiotic stages.

Contig ID	Log_2_ fold change			Annotation
	Dehydration	Inactive Tun	Rehydration	
CL3796Contig1	−6.43	−3.14	−8.53	f-atpase protein 6
CL1Contig11478	−10.03	−3.03	−11.12	cd449245 hypsibius dujardini EST
CL1Contig2465	−2.05	−2.34	−3.05	histone h4
MIRA8_c86294	+∞	+∞	+∞	hypothetical protein
CL1Contig5306	−2.66	−3.07	−2.88	leishmanolysin family protein
MIRA8_rep_c116191	−10.21	−2.05	−6.13	MOB kinase activator 1A
CL1Contig11992	−2.38	−2.28	−2.70	nuclear localization sequence-binding protein
CL1705Contig1	−2.62	−2.94	−3.62	hypothetical protein
MIRA8_c9661	−3.25	−2.80	−2.98	proliferating cell nuclear antigen (PCNA)
MIRA8_c14819	−3.86	−2.83	−3.62	26 S proteasome non-atpase regulatory subunit 2
CL2682Contig1	−2.77	−2.56	−2.72	60 S ribosomal protein l10
CL1372Contig1	−3.02	−2.99	−3.16	60 S ribosomal protein l16
MIRA8_c18411	−3.22	−2.55	−4.00	60 S acidic ribosomal protein p1
MIRA8_c17476	−2.68	−2.61	−3.24	60 S acidic ribosomal protein p2
CL4061Contig1	−2.89	−3.22	−4.85	40 S ribosomal protein s10
CL1Contig7911	−2.73	−2.41	−3.11	40 S ribosomal protein s13
CL1Contig3151	−2.61	−2.28	−3.45	40 S ribosomal protein s19
CL1Contig4733	−2.76	−2.44	−3.54	40 S ribosomal protein s19
CL4670Contig1	−2.65	−2.66	−4.21	40 S ribosomal protein s6
MIRA8_c28015	3.82	2.70	2.45	saxiphilin
MIRA8_c76517	3.60	2.49	2.14	saxiphilin
CL1Contig758	−2.29	−2.25	−2.90	protein transport protein sec61 subunit gamma
MIRA8_c21880	−3.98	−3.21	−3.90	t-complex protein 1 subunit epsilon
MIRA8_c17706	3.41	2.45	2.49	tolloid-like protein 2

(“∞” is used for undetectable expression in one of the samples/lack of fold change).

#### Protein synthesis and degradation

Interestingly among the down-regulated transcripts were many ribosomal proteins. All ribosomes consist of two parts, the large and small subunits. The large 60 S subunit of the eukaryotic ribosome contains three rRNA molecules (25 S, 28 S, and 5 S) and 46 proteins, whereas the small 40 S subunit includes the 18 S rRNA and 33 proteins [67]. Of the ribosomal associated proteins found to be down-regulated in one or more anhydrobiotic stages were: 60 S subunit (l3, l4, l5, l6, l7, l8, l9, l10, l11, l13, l15, l16, l17, l18, l20, l23, l24, l27, l28, l31, l32, l39, l40, p0, p1, p2) and 40 S subunit (s0, s3, s4, s5, s6, s7, s8, s10, s11, s12, s13, s16, s18, s19, s20, s21, s29, s30), which encompassed >50% of all ribosomal proteins (see also Table 3). The present findings seem to be consistent with other research [68] which found that Ha-RPS28, encoding a protein component of the small subunit of cytoplasmic ribosomes, is down-regulated in both seedling roots and shoots of sun flower induced by drought, high salinity, or abscisic acid. Ribosomal proteins are also mainly down-regulated in *Polypedilum vanderplanki* during the process of desiccation [22]. Similarly, it may be part of an adaptive response to maintain ribosomal function in dehydrated cytoplasm. Surveying our results with annotated functions, we identified additionally 33 down-regulated transcripts which are associated the mitotic cell cycle activity (Table S3 in File S5). Out of those, five were down-regulated during dehydration, 20 down-regulated during rehydration (e.g. CEG IDs: KOG0185.11 and KOG0477.14 in Table S3 in File S5), 1 in the inactive tun stage while seven were found to be down-regulated in more than two stages (e.g. CEG ID: KOG1636.6). In contrast, only three up-regulated proteins (Contig IDs: CL1283Contig1, MIRA8_rep_c140926 and MIRA8_rep_c141368) are related to cell cycle/mitotic activity. The previous findings coupled with our data speculate a general reduction in mitotic activity as a consequence of exposure to abiotic stress. Differential expression provides a means for selectively translating specific mRNAs required for the desiccation response as suggested for the insect parasitic nematode *Steinernema carpocapsae* [29]. Apart from ribosomal proteins, another group of proteins associated with the ribosome and protein synthesis were found to be down-regulated in response to dehydration and rehydration. These include elongation factors that are important for delivering transfer RNAs (tRNA) to the ribosome and translation initiation [69]. These down-regulated transcripts belong to eukaryotic elongation factor 1α (de- and rehydration), elongation factor 1γ (during rehydration) and elongation factor 2 (during dehydration and in the inactive tun). The protein transport protein Sec61α together with Sec61β and Sec61γ forms the Sec61 complex of the translocon for ribosome targeted protein synthesis, and two of the subunits (α,γ) were found to be repressed, i.e. Sec61α during rehydration, Sec61γ in all the three anhydrobiotic stages. Also parts of the proteasome machinery were found to be less abundant during anhydrobiosis. These findings accord with our earlier observations [31], [32], which showed apparent tardigrade-specific adaptation of elongation factors on the protein level. Literature has emerged that supports the adaptation role of elongation factors during desiccation stress. For instance, the levels of the translation elongation factor eEF1D, which stimulates the exchange of GDP bound to EF-1α for GTP, are found to be strongly down-regulated in *Zea mays* 2023 genotype [70] after a drought period. In our current study, the only significantly up-regulated transcript of a ribosomal protein was 40 S s2 (during dehydration), although this could be a false positive (chimera, mismapping). Interestingly, many ribosomal proteins are found to be differentially expressed in a nematode species *Plectus murrayi* during desiccation stress [29], and one of them, Pm-rpl-4, is up-regulated. Similar to Pm-rpl-4, 40S s2 might be part of an adaptive response to maintain ribosomal function in dehydrated cytoplasm.

#### Cytoskeleton

Our results imply remodelling of the cytoskeleton taking place during anhydrobiosis. Cytoskeletal components such as beta actin (rehydration) and beta tubulin (de- and rehydration) were found to be down-regulated, whereas their associated protein cofilin was up-regulated in response to dehydration and rehydration. Cofilin binds and disassembles actin filaments. Also the F-actin capping protein, a dimer of unrelated proteins (α, β) that blocks the ends of polymerizing actin filaments, was found to be differentially expressed. The β-unit was up-regulated during dehydration and the α-unit down-regulated during rehydration. There are similarities between the expression of cytoskeletal components in this study and those described by Tyson et al. [71]. Their data provided the evidence on the elevated expression of TUB3 and SRO9 in *Panagrolaimus superbus*, a free-living anhydrobiotic nematode [71] subject to environmental desiccation. TUB3 encoding α-tubulin is involved in mitotic segregation and nuclear migration, whereas SRO9P associating with translating ribosomes is important in the organization of actin filaments. Changes in microtubule dynamics have been found to occur during osmotic stress in *Brassica napus* during desiccation [72]. Verification of transcriptome data from a bdelloid species *Adineta ricciae* reveals that bdelloid species contain a dozens of different copies of α-tubulin crucial to surviving desiccation at all life stages [72]. As the cells of anhydrobiotic animals undergo large volume changes during desiccation and subsequent rehydration, the cytoskeleton has probably to adapt accordingly.

#### DNA replication

Previously identified differential transcripts fit well into the picture of inhibited cell division. The cloning of replication factor C subunit 2 by means of representational difference analysis has been previously reported [73]. The replication factor C is a direct binding partner of proliferating cell nuclear antigen (PCNA). PCNA was found to be down-regulated in all three anhydrobiotic stages (Table 3). Interestingly, we found two types of histone encoding transcripts (H3.3 and H4) repressed compared with the active stage. Histones are the main protein component of the chromatin and important for DNA packing and gene expression regulation. One group includes canonical core histones (H2A, H2B, H3 and H4) and a linker histone H1 which are replication-dependent. In other words, they are only transiently expressed during the S-phase (synthesis phase) of the cell cycle. The other group consists of replication independent histones such as H3.3, H2A.Z (H2Av in *D. melanogaster*), CENPA, macro-H2A and histone H1.0. One peculiarity of replication dependent histones is the fact that they are generally considered not to be polyadenlyated in higher eukaryotes [74]. In principle the sequencing approach employed in this study should be agnostic to RNAs lacking polyA tails. There have been several reports of alternatively processed histone mRNAs containing polyA tails, some of them using similar sequencing approaches such as Poly(A) Site Sequencing (PAS-Seq) [75]–[77]. Therefore, some plasticity in histone mRNA processing may produce polyadenlyated transcripts under particular circumstances in different organisms. This might explain the identification of histone H4 along with the polyadenylated histone H3.3 in this study.

#### DNA repair

Desiccation could damage molecules, including DNA. The role of DNA repair in desiccation-tolerant plants and prokaryotes has been underscored in several studies [78], [79]. A time-dependent increase in DNA damage has been evidenced in the anhydrobiotic tardigrade *Paramacrobiotus richtersi* [80]. Our functional annotations suggest that the *M. tardigradum* genome had genes encoding desiccation-responsive proteins (e.g. DESeq IDs: 7428, 15192 and 15186 in Table S4 in File S5, see also Files S2, S3, S4) that took part in different DNA repair mechanisms, including translesion synthesis, nucleotide excision repair, base excision repair, mismatch repair and photoactivation (Table S4 in File S5). Of notice, polyubiquitin gene *UBB* (DESeq ID: 18963 in Table S4 in File S5, see also File S4) was found to be up-regulated in response to rehydration. This finding supports previous research into the involvement of ubiquitin in vegetative desiccation tolerance [79]. In both the modified desiccation-tolerant grass *Sporobolus stapfianus* and the fully desiccation-tolerant moss *Tortula ruralis* [79], O'Mahony and Oliver found accumulation of ubiquitin transcripts in response to drying and during the rehydration phase. Their data indicate that ubiquitin transcripts, which can be translated upon rehydration, enable the cellular repair via ubiquitin-mediated protein degradation. Moreover, we found several transcripts whose read abundances were higher during rehydration than in the dehyrdation stage. It has been proposed that a desiccation-tolerant plant would be able to mobilize its DNA repair mechanism to combat its desiccation damage [81]. Similarly, it is temping to surmise a mobilisation of DNA repair mechanism upon rehydration in *M. tardigradum*.

#### Up-regulated transcripts

Among the few induced transcripts were some from the small heat-shock family of proteins. The small heat shock/alpha-crystallin protein p26 has been extensively studied in anhydrobiotic brine shrimp cysts, where it accumulates and can make up 10–15% of the non-yolk protein [82]–[84]. Similar patterns of up-regulation of small Hsps have also been found in anhydrobiotic chironomid larvae [85]. We observed an up-regulation of Hsp27 and Hsp30c during de- and rehydration. Hsp27 and Hsp30c are supposed to be involved in cell cycle and differentiation [86], [87]. A direct evidence on desiccation responsive Hsp27 in other species has been lacking. However, up-regulation of small heat shock/alpha-crystallin protein *Pv-hsp20*, a homolog of Hsp27, has been observed at the early stage of desication in chironomid larvae [85]. The induction of the both transcripts was more pronounced *during rehydration* compared to dehydration. These findings seem to accord the observation of et al. who found an mRNA abundance for *Pv-hsp20* remains high even 24 h after recovery from desiccation. Hsp30 has been found to be pronouncedly induced in hepatopancreas of *Sphincterochila zonata* [86] exposed to normothermic desiccation, suggesting an important role of this protein in cellular processes following desiccation. These findings further support the idea of Rinehart and Denlinger [88] who suggested that in the flesh fly *Sarcophaga crassipalpis*, there are the constitutive and inducible groups of Hsps with different functions in response to desiccation and rehydration. Desiccation responsive Hsps are believed to bind to denatured proteins caused by water loss and inhibit aggregation, whereas rehydration-elicited up-regulation of Hsps helps to reinitiate productive protein folding pathways and re-establish membrane fluidity [88]. Since the rapid uptake of water has also the potential to cause cell damage during rehydration [88], Hsps seem to play an important role (e.g. Re-initiating the normal protein synthesis) during stress recovery. Furthermore some protease inhibitors were also induced, which might either provide a self-protective mechanism against endogenous proteases or a defence against microbial degradation during the time spent as an inactive tun. Some of the remaining up-regulated transcripts belong to hypothetical proteins with rather good conservation in many insect species but without any known function.

## Conclusions

The interesting patterns of differentially expressed transcripts which we have found include the down-regulation of several proteins of the DNA replication and translational machinery, protein degradation and the up-regulation of heat shock proteins Hsp27 and Hsp30c, and the polyubiququin protein (*UBB*) related to DNA repair. Most of the transcripts identified to be differentially expressed had distinct cellular functions. The results suggest that the concerted molecular modification contributes to a protection mechanism, permitting extreme forms of ametabolic states in tardigrades. In addition to the rehydration-induced up-regulation of polyubiququin, several genes involved in DNA repair have been found to be more pronounced in the rehydration stage than in the dehydration stage, potentially implying the mobilization of cellular repair mechanisms in *M. tardigradum* upon rehydration. Hence, *M. tardugradum* appears to adopt a tolerance strategy that combines a constitutive protection system with a rehydration-inducible recovery mechanism. Our findings shed light on the molecular adaptations required for *M. tardigradum* to combat desiccation stress.

Many of the differentially expressed transcripts identified in this study are clearly basal and highly conserved cellular components that govern global metabolism of the organism and have already been described in a wide range of animals to be involved during metabolic rate depression induced by various factors such as high and low temperature, oxygen deprivation, food restriction and water limitation. The current study has only limited data on rehydration (1 h hydration) and may not reflect the full stress recovery. Our finding on the molecular repair is still preliminary. Further research with more rehydration data sampled over an extension of time might help us to establish a greater degree of accuracy on this matter. Note that changes in mRNA accumulation may not necessarily correlate with those at the protein activity level. More research on this topic using other complementary techniques (e.g. transcriptional regulatory networks) needs to be undertaken before the mechanisms regarding stage transitions of anhydrobiosis in *M. tardigradum* is more clearly understood.

## Materials and Methods

### Animal culture

*M. tardigradum* was cultured on petri-dishes (ø 9.4 cm) with a layer of agarose (3%) (peqGOLD Universal Agarose, PEQLAB, Erlangen Germany) covered with a thin layer of Volvic water (Danone Waters, Wiesbaden, Germany) at 20°C. The animals were fed bdelloid rotifers, *Philodina citrina* Ehrenberg, 1832, which had been raised on the green algae *Chlorogonium elongatum* (P.A. Dangeard) Francé 1897. Before RNA extraction the animals were starved for two days to avoid contamination. After repeated washing with Volvic water, 200 animals were transferred into 1.5 ml tubes and residual water was removed using a micropipette. The open tubes were then exposed to 85% relative humidity (RH) in a small chamber containing a saturated solution of KCl (Roth, Karlsruhe, Germany). Animals for the dehydrated stage were collected once head and legs were retracted and tun formation was complete. For completely desiccated inactive tuns the animals were further dehydrated for 24 h and then dried at 35% RH for an additional 48 h in a chamber containing a saturated solution of MgCl2 (Roth). Rehydration was monitored using a stereo-microscope and animals failing to rehydrate after 1 h or showing abnormal morphology would be discarded. In the current study, 200 animals were used for each anhydrobiotic state. Four different stages investigated are: active (i.e. active animals used as control), dehydration, inactive tun (72 h of dehydration) and rehydration stage (1 h of rehydration).

### Preprocessing of sequence datasets

#### Sanger clone data

Sanger clones were processed according to the protocol described in Beisser et al. [89]. Briefly, this included base calling and initial quality trimming Sanger sequencing traces using Phred (version 0.071220.c) [90], masking adapters and vector sequences using cross_match version 1.090518 software (source: http://www.phrap.org webcite), and polyA-tail removal using SnoWhite (version 1.1.3) [91].

#### 454 pyrosequencing data

For 454 sequencing cDNA libraries generated from active, dehydration, inactive tun and rehydration stages were normalized using the duplex specific nuclease method (Trimmer-direct kit, Evrogen, Moscow, Russia), following manufacturers' recommendations [92]. The separately normalized cDNA libraries were pooled and sequenced using FLX Titanium chemistry at GATC Biotech. PolyA trimming and adaptor removal was performed using SnoWhite as for the Sanger data. Sequence reads have been deposited to the NCBI sequence read archive (SRA) under the study accession number SRA020123.

#### Illumina data

Illumina sequencing was performed at GATC Biotech (Konstanz, Germany) following a custom proprietary library preparation protocol. Agarose gel electrophoresis was used to judge the integrity and overall quality of total RNA samples by inspection of the 28 S and 18 S rRNA bands (Figure S1 in File S5). From the total RNA samples of all four stages, cDNA was synthesized using an oligo(dT)-linker primer (5′-GCCTGTCACTCACTGCGA-T25-NV-3′) and M-MLV H- reverse transcriptase for first strand synthesis. The reaction conditions were chosen such that the length of first-strand cDNA was limited to 100–500 nucleotides. After second strand synthesis and sequencing adaptor ligation the cDNA was PCR amplified in 19 cycles using a high fidelity DNA polymerase. cDNAs in the size range of 200–350 bp were eluted from preparative agarose gel and each of the samples was sequenced on a single lane on an Illumina Genome Analyzer II for 36 cycles. Illumina sequence data was analysed using SolexaQA version 2.2 software [93] (Figure S2 in File S5). The overall error probabilities are in the range expected for this sequencing chemistry. PolyA-only reads or where the majority of the read consisted of adenines were trimmed using cutadapt version 1.1 software [94]. Reads shorter than 25 bases after trimming were discarded. To extract the reads from the sff files and store them into fasta, xml or caf text files, sff_extract version 0.3.0 software (source: http://bioinf.comav.upv.es/sff_extract/) was used. For the error-correction of Illumina datasets, Reptile (version 1.1) [43] was used. Sequence reads are available at the SRA of NCBI with the accession numbers SRX426237 (active), SRX426238 (inactive tun), SRX426239 (dehydration) and SRX426240 (rehydration) associated with the study accession number SRA020123.

### *De novo* assembly

#### Assembly algorithms

For performing hybrid transcriptome assemblies, it has been proposed to combine different assemblers to accommodate to algorithmic differences and different data types [95]. There are two main assembly algorithms, OLC [41] and DBG assemblers [39], [40]. OLC assemblers such as MIRA [41] and Newbler (Roche/454 assembler) take advantage of the whole sequence read and are more robust to low quality sequences. However, these assemblers do not scale very well computationally as each read has to be compared against all others in some way to find overlaps for assembly. In contrast, DBG assemblers adopt a fundamentally different approach to sequence assembly, where reads are not treated as sequence strings as a whole, but are broken up into fixed words of length k (k-mers) and assembled as k-1 overlaps. This approach scales well for large data amounts as identical k-mers are collapsed when computed from the original reads. High coverage transcripts will assemble well with longer k-mers, whereas low coverage transcripts will be fragmented in lack of sufficient overlaps. In the current study, we used a DBG assembler to generate longer contigs from short high-throughput sequencing data as the vast amount of sequence data typically cannot be handled by assemblers that are optimized for Sanger and low coverage 454 data. For assembling the original longer reads together with the resulting contigs as pseudo-reads, we employed an OLC assembler.

#### Error correction and pre-assembly of short read data

In contrast to Sanger sequencing, NGS suffers from a higher error rate. The “flow” based sequencers such as 454 or Ion Torrent typically exhibit insertion/deletion errors [96], [97] due to jointly sequence runs of the same bases (homopolymers), whereas Illumina sequencers have a sequence specific error profile [98]. As DBG assemblers are highly susceptible to sequencing errors, the Illumina dataset was error-corrected prior to assembly using Reptile version 1.1 software [43]. The Reptile error-corrected Illumina sequences were then assembled using ABySS (version 1.2.2) [42] with standard parameters and a k-mer parameter from 20 to 35. Contigs which are less than 100 bases were discarded. The 454 dataset plus the Reptile corrected Illumina reads were error-corrected using Hybrid-SHREC [44] (version 1.0) since Hybrid-SHREC is a suffix trie based error correction tool that can handle reads from all common sequencing platforms and corrects substitution as well as insertion/deletion errors [99]. For the ABySS Illumina+454 assemblies some additional parameters of ABySS were used (see File S6). To generate a non-redundant dataset that contains only unique contigs from all ABySS assemblies, the single assemblies were combined using the clustering tool CD-HIT-EST (version 4.0) [45].

#### Hybrid assembly

For merging the assemblies, all contigs with at least 100 bases derived from assemblies with a k-mer from 20 to 35 were collapsed at 100% identity. The hybrid assembly was performed using MIRA assembler (version 3.2.1) [1]. This approach combined the Sanger, 454 reads plus the two pre-assembled datasets (ABySS assemblies) into a single assembly. Further details on the parameter settings for those programs can be found in File S6.

#### Library-less repeat masking and final clustering and assembly

Subsequent to the hybrid assembly with MIRA, the sequences were clustered and assembled using TGICL pipeline (version 2.1) [46] to produce more complete consensus sequences. EST clustering and assembly depends on identifying matching sequences by similarity searches. Here repetitive sequences can give rise to inflated clusters because sequence reads from different transcripts may be clustered together and subsequent assembly might result in mis-assemblies. A library-less approach [49] was adopted in the current study because repeat libraries have little value across distantly related species and there are no tardigrade repeat libraries available either. Prior to clustering and assembly with TGICL, the contigs were soft-masked (lower case) using RBR [47], [48] (version 0.8.6).

#### Scaffolding

Scaffolding was performed via a scaffolding translation mapping (STM) approach that uses protein coding information in fragmented transcriptome assemblies to generate scaffolds by leveraging proteome data, ideally from a related organism [51]. To scaffold the remaining contigs into more contiguous sequences, the MIRA assembly contigs and singletons were first reduced in complexity using CD-HIT-EST at 95% similarity (removing mostly short redundant contigs with sequencing errors). The *D. melanogaster* gene translations for release 5.37 were downloaded from FlyBase [100]. The protein translations were made non-redundant using CD-HIT at 100% identity. BLASTx [101] (version 2.2.18) was used to map the *M. tardigradum* sequence onto the *D. melanogaster* non-redundant proteome. To avoid scaffolding onto low complexity parts of unrelated proteins, the reference *D. melanogaster* proteome was soft-masked using segmasker included as part of the BLAST+ distribution (version 2.2.25) [101]. The code of STM was modified to allow DNA ambiguity codes other than “N” in contigs as produced by the MIRA assembler.

### Assessing quality of *de novo* transcriptome

In order to assess completeness of the transcriptome assembly, the CEGMA pipeline [48] (version 2.4) was used. Additionally, est2assembly [52] (version 1.13) was employed to compare the transcriptome assembly with publicly available sequence datasets. The genome and proteome datasets for reference organisms used in this study were downloaded from ENSEMBL Metazoa (release 20) [49].

### BLAST annotation of transcripts

To annotate the transcripts, we used Blast2GO software (version 2.1.0) [55]. The assembled transcripts were successive searched against various databases. Only sequences without match were carried over to the next search. The search order was LSURef_SILVA, SSURef_SILVA, SwissProt, NCBI nr and *H. dujardini* ESTs. The SSURef_SILVA database was clustered using CD-HIT-EST (version 4.0) at 95% identity prior to being searched against. This was done to save computation time as our goal was not to accurately assign species to reads matching ribosomal sequences but to filter them.

### Mapping of Illumina reads and quantification

BWA [58] (version 0.6.2) and Stampy [59] (version 1.0.21) aligners were used to map the Illumina reads onto the transcriptome assembly. The BWA is a fast aligner optimized for near-identical matches, especially short Illumina sequences [58]. By comparison, Stampy is a seed based aligner that is more specific, but slower [59]. A combination of these two aligners has been chosen in this study, since neither the input reads for mapping nor the reference could be expected to be error free. Especially as the reference was mostly generated from 454 data that has a completely different error model from the Illumina data that was being mapped. Stampy can utilize BWA as a pre-mapper to find almost identical hits and re-map the remaining reads for better sensitivity, thereby combining the advantages of both (An overview of the mapping performance of the two used aligners is given in Table S5 in File S5). Specifically, read mapping was performed using BWA with a minimum read quality trimming of 10. Equally best hits (multi-hits) were written as separate entries into the SAM file from the “XA” tag using the “xa2multi.pl” script. A maximum of three possible alternative maps were reported and reads that mapped to more than three positions were randomly assigned to one of them. Mapping using Stampy with BWA in hybrid mode was performed using identical BWA parameter and the “sensitive” option of Stampy. All other parameters were set to default. All reads with a mapping quality of 20 or greater were retained and also all multi-maps (MapQ 0).

Counts for each stage were generated using SAMtools (version 0.1.19) [102]. Contigs without counts in any of the stages were removed before further analysis. The counts for each contig were loaded into DESeq [95] (version 1.10.1) and independent filtering as well as differential gene expression analysis was performed according to the DESeq manual for experiments without replicates using all available stages as pseudo-replicates for variance estimation. For quantification, potential PCR duplicates were not removed as the reads cluster at the 3′ end of the transcripts. Identical reads that map to identical positions are sometimes removed to mitigate the effect of PCR library amplification bias. But there are also arguments against this practice. For instance, such a removal would reduce dynamic range due to mapping saturation as not all duplicate mappings are actually PCR derived. Considering a 300 bp window there is only a limited number of mapping positions. So any coverage exceeding this value would be identified as a PCR duplicate in this study.

## Supporting Information

File S1 **Predicted CEGs present in the** ***M. tardigradum*** **assembly and their annotation.**
(XLS)

File S2 **Transcripts differentially expressed between the active and dehydration stages and their annotation.**
(XLS)

File S3 **Transcripts differentially expressed between the active and inactive tun stages and their annotation.**
(XLS)

File S4 **Transcripts differentially expressed between the active and rehydration stages and their annotation.**
(XLS)

File S5 **Supplementary tables and figures.**
(DOC)

File S6 **Programs used in the study and their parameter settings.**
(DOC)
