# Supplementary material for: Towards Decrypting Cryptobiosis—Analyzing Anhydrobiosis in the Tardigrade Milnesium tardigradum Using Transcriptome Sequencing
Source: PLoS One. 2014 Mar 20;9(3):e92663. doi: 10.1371/journal.pone.0092663 (PMC3961413; doi:10.1371/journal.pone.0092663)
Supplement: File S5 — Supplementary tables and figures. (DOC) [file pone.0092663.s005.doc]

# Supplementary Tables and Figures

## Prediction of core eukaryotic genes (CEGs)

According to CEGMA [1], core eukaryotic genes (CEGs) are classified as four groups based on the average degree of conservation. Group 1 represents the least conserved genes of the reference set 248 CEGs, with the conservation degree increasing in subsequent groups through to group 4. A detailed information on the prediction of the presence of CEGs in the *M. tardigradum* and the reference genomes are summarized in Table S1.

## Table S1 - Prediction of the 248 CEGMA core eukaryotic genes in *M. tardigradum* and reference organisms

| Organisms | Group 1 | | Group 2 | | Group 3 | | Group 4 | |
| --- | --- | --- | --- | --- | --- | --- | --- | --- |
|  | Compl. | Partial | Compl. | Partial | Compl. | Partial | Compl. | Partial |
| *M. tardigradum* | 72.73 | 87.88 | 85.71 | 92.86 | 81.97 | 93.44 | 90.77 | 100.00 |
| *An. gambiae* | 93.94 | 98.48 | 96.43 | 98.21 | 93.44 | 96.72 | 100.00 | 100.00 |
| *C.elegans* | 92.42 | 96.97 | 91.07 | 92.86 | 91.80 | 93.44 | 81.54 | 83.08 |
| *D. pulex* | 95.45 | 96.97 | 98.21 | 98.21 | 96.72 | 98.36 | 95.38 | 96.92 |
| *D. melanogaster* | 96.97 | 98.48 | 96.43 | 96.43 | 96.72 | 100.00 | 98.46 | 100.00 |
| *I. scapularis* | 27.27 | 71.21 | 50.00 | 82.14 | 39.34 | 83.61 | 53.85 | 84.62 |
| *P. pacificus* | 77.27 | 90.91 | 89.29 | 92.86 | 81.97 | 90.16 | 90.77 | 98.46 |
| *S. maritima* | 95.45 | 98.48 | 96.43 | 96.43 | 98.36 | 100.00 | 98.46 | 98.46 |
| *T. urticae* | 92.42 | 95.45 | 94.64 | 100.00 | 96.72 | 98.36 | 96.92 | 98.46 |

(“Group 1 – Group 4” - CEGs groups with increasing degree of conservation from group 1 through to group 4 as classified in [1]; “Compl.” - complete)

## CEGs differentially expressed in anhydrobiotic stages

Nine CEGs were found to be differentially expressed during anhydrobiosis. Most of them were rehydration-responsive, and two of them (KOG IDs: 1636.6 and 3449.1) were responsive in all the three anhydrobiotic stages.

## Table S2 - Core eukaryotic genes differentially expressed in anhydrobiotic stages.

| KOG  ID | Description | Stage | Swiss-Prot  Acc  Number | NCBI  GI  Number | E-Value |
| --- | --- | --- | --- | --- | --- |
| 0073.2 | arl2_human ame: full=adp-ribosylation factor-like protein 2 | D | P36404.4 | 116241255 | 2.5503E-71 |
| 0185.11 | psb4_mouse ame: full=proteasome subunit beta type-4 ame: full=low molecular mass protein 3 ame: full=macropain beta chain ame: full=multicatalytic endopeptidase complex beta chain ame: full=proteasome beta chain ame: full=proteasome chain 3 flags: precursor | R | P99026.1 | 3914439 | 3.48676E-60 |
| 0477.14 | mcm2_human ame: full=dna replication licensing factor mcm2 ame: full=minichromosome maintenance protein 2 homolog ame: full=nuclear protein bm28 | R | P49736.4 | 41019490 | 7.92521E-165 |
| 1268.11 | gfpt1_human ame: full=glutamine--fructose-6-phosphate aminotransferase | R | Q06210.3 | 30923274 | 3.32324E-76 |
| 1349.2 | gpi8_mouse ame: full=gpi-anchor transamidase short=gpi transamidase ame: full=phosphatidylinositol-glycan biosynthesis class k protein short=pig-k flags: precursor | R | Q9CXY9.2 | 51338819 | 1.54798E-113 |
| 1636.6 | pcna2_dauca ame: full=proliferating cell nuclear antigen large form short=pcna ame: full=cyclin | D,R,T | Q00265.1 | 129690 | 8.78572E-71 |
| 2529.7 | cbf5_klula ame: full=centromere microtubule-binding protein cbf5 ame: full=centromere-binding factor 5 ame: full=h aca snornp protein cbf5 ame: full=small nucleolar rnp protein cbf5 | R | O13473.1 | 3023443 | 6.18692E-172 |
| 2738.2 | ampm1_xentr ame: full=methionine aminopeptidase 1 short=map 1 short= 1 ame: full=peptidase m 1 | R | Q5I0A0.1 | 82179280 | 6.66464E-73 |
| 3449.1 | rla2_cryst ame: full=60s acidic ribosomal protein p2 | D,R | O61463.1 | 3914777 | 8.95751E-14 |

(“R” – rehydration; “D” - dehydration; “T” - inactive tun)

## Transcripts involved in mitotic activity

In addition to ribosome proteins such as 60s ribosomal protein l24 and 40s ribosomal protein s6-b, we found other differentially expressed transcripts related to mitotic activity (Table S3). For instance, mitotic cyclins were differentially expressed in the anhydrobiotic states. Alpha-tubulin was responsive to both dehydration and rehydration. Beta-tubulins of the mitotic spindle except for beta 2b chain were down-regulated during rehydration.

## Table S3 - Differentially expressed transcripts with annotated functions involved in mitotic cell cycle.

| Contig  ID | Description | Expression  level | Anhydrobotic  stage | CEGs ID |
| --- | --- | --- | --- | --- |
| CL1Contig931 | ccna2_chick ame: full=cyclin-a2 short=cyclin-a | down | rehydration |  |
| CL1Contig2019 | psd13_bovin ame: full=26s proteasome non-atpase regulatory subunit 13 ame: full=26s proteasome regulatory subunit rpn9 ame: full=26s proteasome regulatory subunit s11 ame: full=26s proteasome regulatory subunit | down | de- and rehydration |  |
| CL1Contig2791 | psb4_mouse ame: full=proteasome subunit beta type-4 short=proteasome beta chain ame: full=low molecular mass protein 3 ame: full=macropain beta chain ame: full=multicatalytic endopeptidase complex beta chain ame: full=proteasome chain 3 flags: precursor | down | rehydration | KOG0185.11 |
| CL1Contig3523 | tbb_copc7 ame: full=tubulin beta chain ame: full=beta-tubulin | down | rehydration |  |
| CL1Contig3838 | ago11_orysj ame: full=protein argonaute 11 short= 11 | down | rehydration |  |
| CL1Contig7574 | csn2_rat ame: full=cop9 signalosome complex subunit 2 short=sgn2 short=signalosome subunit 2 ame: full=alien homolog ame: full=jab1 containing signalosome subunit 2 ame: full=thyroid receptor-interacting protein 15 short=tr-interacting protein 15 short=trip-15 | down | inactive tun,  rehydration |  |
| CL1Contig11839 | tba1_homam ame: full=tubulin alpha-1 chain ame: full=alpha-i tubulin | down | de- and rehydration |  |
| CL1Contig12123 | tbb2b_human ame: full=tubulin beta-2b chain | down | de- and rehydration |  |
| CL912Contig1 | tfdp2_human ame: full=transcription factor dp-2 ame: full=e2f dimerization partner 2 | down | rehydration |  |
| CL1221Contig1 | smc4_arath ame: full=structural maintenance of chromosomes protein 4 short= 4 short=smc protein 4 short=smc-4 ame: full=chromosome-associated protein c short= -c | down | de- and rehydration |  |
| CL1277Contig1 | rpn6_schpo ame: full=probable 26s proteasome regulatory subunit rpn6 | down | rehydration |  |
| CL1283Contig1 | tbcb_human ame: full=tubulin-folding cofactor b ame: full=cytoskeleton-associated protein 1 ame: full=cytoskeleton-associated protein ckapi ame: full=tubulin-specific chaperone b | up | dehydration |  |
| CL1843Contig2 | psb5_dicdi ame: full=proteasome subunit beta type-5 flags: precursor | down | rehydration |  |
| CL1908Contig1 | mcm2_human ame: full=dna replication licensing factor mcm2 ame: full=minichromosome maintenance protein 2 homolog ame: full=nuclear protein bm28 | down | rehydration | KOG0477.14 |
| CL1919Contig1 | if4e1_maize ame: full=eukaryotic translation initiation factor 4e-1 short=eif-4e-1 short=eif4e-1 ame: full=eif-4f 25 kda subunit ame: full=eif-4f p26 subunit ame: full=mrna cap-binding protein | down | rehydration |  |
|  |  |  |  |  |
| CL3214Contig1 | psa1_bovin ame: full=proteasome subunit alpha type-1 | down | rehydration |  |
|  |  |  |  |  |
| CL4905Contig1 | dpola_xenla ame: full=dna polymerase alpha catalytic subunit ame: full=dna polymerase alpha catalytic subunit p180 | down | rehydration |  |
| CL5442Contig1 | psmd2_rat ame: full=26s proteasome non-atpase regulatory subunit 2 | down | rehydration |  |
| MIRA8_c2092 | tfpi1_rat ame: full=tissue factor pathway inhibitor short=tfpi ame: full=extrinsic pathway inhibitor short=epi ame: full=lipoprotein-associated coagulation inhibitor short=laci flags: precursor | down | dehydration |  |
| MIRA8_c3705 | psmd2_rat ame: full=26s proteasome non-atpase regulatory subunit 2 | down | de- and rehydration |  |
| MIRA8_c4184 | tbb_lymst ame: full=tubulin beta chain ame: full=beta-tubulin | down | rehydration |  |
| MIRA8_c7934 | psd12_bovin ame: full=26s proteasome non-atpase regulatory subunit 12 ame: full=26s proteasome regulatory subunit rpn5 | down | rehydration |  |
| MIRA8_c9661 | pcna2_dauca ame: full=proliferating cell nuclear antigen large form short=pcna ame: full=cyclin | down | inactive tun,  de- and rehydration | KOG1636.6 |
| MIRA8_c14819 | psmd2_rat ame: full=26s proteasome non-atpase regulatory subunit 2 | down | inactive tun,  de- and rehydration |  |
|  |  |  |  |  |
| MIRA8_c15193 | erf3b_mouse ame: full=eukaryotic peptide chain release factor gtp-binding subunit erf3b short=eukaryotic peptide chain release factor subunit 3b short=erf3b ame: full=g1 to s phase transition protein 2 homolog | down | dehydration |  |
| MIRA8_c19323 | cdc42_macfa ame: full=cell division control protein 42 homolog flags: precursor | down | rehydration |  |
| MIRA8_c16091 | smc4_arath ame: full=structural maintenance of chromosomes protein 4 short= 4 short=smc protein 4 short=smc-4 ame: full=chromosome-associated protein c short= -c | down | rehydration |  |
| MIRA8_c24506 | psb6_dicdi ame: full=proteasome subunit beta type-6 ame: full=differentiation-associated proteasome subunit 1 short=daps-1 flags: precursor | down | rehydration |  |
| MIRA8_c25263 | pcna_soybn ame: full=proliferating cell nuclear antigen short=pcna ame: full=cyclin | down | de- and rehydration |  |
| MIRA8_c34704 | gsp1_canal ame: full=gtp-binding nuclear protein gsp1 ran | down | dehydration |  |
| MIRA8_c63004 | tbb4_chick ame: full=tubulin beta-4 chain ame: full=beta-tubulin class-iii | down | rehydration |  |
| MIRA8_c70011 | gsp1_ashgo ame: full=gtp-binding nuclear protein gsp1 ran | down | inactive tun |  |
| MIRA8_rep_c115013 | dyl1_drome ame: full=dynein light chain cytoplasmic ame: full=8 kda dynein light chain ame: full=cut up protein | down | rehydration |  |
| MIRA8_rep_c140926 | ubb_sheep ame: full=polyubiquitin-b contains: ame: full=ubiquitin-related contains: ame: full=ubiquitin flags: precursor | up | rehydration |  |
| MIRA8_rep_c141368 | ubiqh_dicdi ame: full=polyubiquitin-h contains: ame: full=ubiquitin | up | rehydration |  |

(“down” – down-regulated; “up” - up-regulated)

## Transcripts related to cellular repair

We found 14 differentially expressed transcripts which are associated with DNA repair. All the 14 were rehydration responsive. And one of them (DESeq ID: 18963) was up-regulated during rehydration.

## Table S4 - Transcripts of *M. tardigradum* related to DNA repair mechanisms.

| DESeq | References ID | Read Abundance | | Repair Mechanisms |
| --- | --- | --- | --- | --- |
| ID |  | Dehydration | Rehydration |  |
| 5562 | CL4905Contig1 | - | 24.759 | DNA synthesis involved in DNA repair |
| 7356 | CL2301Contig1 | - | 6.830 | photorespiration |
| 7428 | CL1Contig6866 | 0 | 0.854 | rhodopsin mediated phototransduction; G-protein coupled photoreceptor activity; photoreceptor inner segment |
| 12192 | MIRA8_c9661 | 41.922 | 50.372 | error-prone translesion synthesis;, mismatch base repair; coupled nucleotide-excision repair; base-excision repair |
| 12195 | MIRA8_c25263 | 36.454 | 50.372 | translesion synthesis; mismatch repair; nucleotide-excision repair; base-excision repair |
| 14730 | MIRA8_c12997 | - | 46.103 | DNA repair |
| 15166 | MIRA8_c28808 | - | 8.538 | transcription-coupled nucleotide-excision repair |
| 15605 | MIRA8_c11407 | - | 31.589 | base-excision repair; negative-regulation of DNA repair |
| 15609 | CL1226Contig1 | - | 15.368 | base-excision repair; negative-regulation of DNA repair |
| 16632 | CL1221Contig1 | 31.897 | 11.099 | DNA repair |
| 16633 | MIRA8_c16091 | - | 11.953 | DNA repair |
| 17999 | MIRA8_c19885 | - | 23.905 | double-strand break repair |
| 18963 | MIRA8_rep_c140926 | - | 4378.938 | DNA repair |
| 34735 | MIRA8_c85638 | - | 40.981 | DNA mismatch repair |

(“-” - non-responsive)

## Mapping performance of aligners

A comparison between BWA [2] plus Stampy [3] and BWA solely is given in Table S5. The data in columns 3 and 4 refer to the fractions of reads mapped in the four datasets using BWA alone and in combination with Stampy, respectively. Comparison between the proportions of mapped reads by two approaches suggests that around 10% more reads could be mapped by BWA plus Stampy than BWA solely.

## Table S5 - Fractions of reads mapped in the four datasets using BWA alone and in combination with Stampy.

| Dataset | # reads | BWA | BWA+Stampy |
| --- | --- | --- | --- |
| active | 6,983,668 | 67.78% | 78.51% |
| dehydration | 7,901,186 | 80.75% | 91.69% |
| rehydration | 7,753,843 | 86.07% | 95.10% |
| inactive | 7,304,785 | 74.32% | 86.76% |

## RNA analysis

Figure S1 presents the result of RNA analysis using agarose gel electrophoresis. For each stage two separate RNA extractions of 200 animals after sonication are shown. The 28S and 18s rRNA bands are clearly visible and RNA yield and integrity are reproducible between technical replicates.


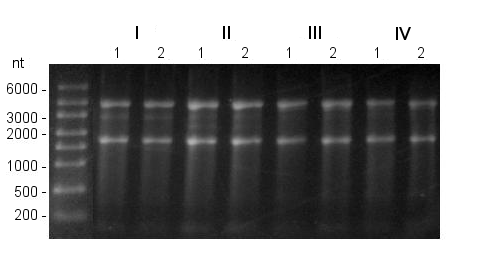


Figure S1 - Total RNA analysis using agarose gel electrophoresis. RNA extractions from four stages: active (I), dehydration (II), inactive tun (III) and rehydration (IV).

## Quality assessment of Illumina sequencing data

Illumina sequence data was analyzed using SolexaQA [4]. The SolexaQA graphical output is presented in Figure S2. The increased error rate at base position 9 of tile 18 in the Mt_ACT2 dataset (active stage) is probably due to air bubble formation on the sequencing chip. As a result, the dataset contained a 4.5 fold (160,167) increased number of reads with an uncalled base (“N”) at this position as compared to the adjacent positions 8 (35,747) and 10 (35,954) of that tile.


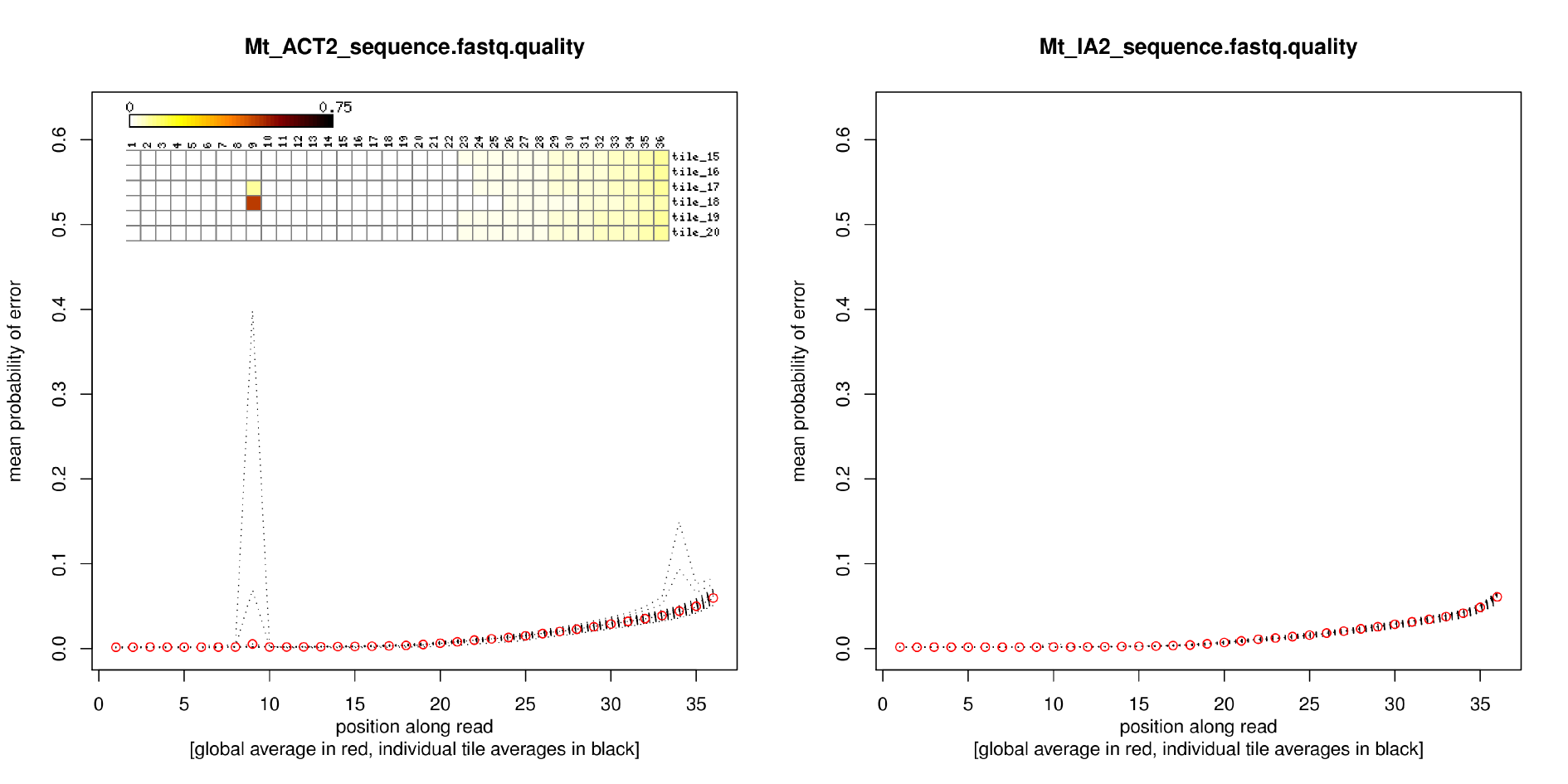


Figure S2: SolexaQA quality assessment of Illumina sequencing.

Shown are the per cycle error rate exemplary for the datasets Mt_ACT2 (active stage) and Mt_IA2 (rehydration). The inset in Mt_ACT2 depicts the local increase of error probability in tile_18 of the chip.

# ****References****

1. **Parra G, Bradnam K, Ning Z, Keane T, Korf I (2009) Assessing the gene space in draft genomes. Nucleic Acids Research 37(1): 298-297**.
2. Li H, Durbin R (2009) Fast and accurate short read alignment with Burrows-Wheeler transform. Bioinformatics 25: 1754-176.
3. **Lunter G, Goodson M (2011) Stampy: a statistical algorithm for sensitive and fast mapping of Illumina sequence reads. Genome Res 21: 936-939.**
4. **Cox MP, Peterson DA, Biggs PJ (2010) SolexaQA: At-a-glance quality assessment of Illumina second-generation sequencing data. BMC bioinformatics11: 485.**
