# Supplementary material for: Towards Decrypting Cryptobiosis—Analyzing Anhydrobiosis in the Tardigrade Milnesium tardigradum Using Transcriptome Sequencing
Source: PLoS One. 2014 Mar 20;9(3):e92663. doi: 10.1371/journal.pone.0092663 (PMC3961413; doi:10.1371/journal.pone.0092663)
Supplement: File S6 — Programs used in the study and their parameter settings. (DOC) [file pone.0092663.s006.doc]

## ****Program parameters****

Below is a list of programs together with their source and version information as well as parameter settings used in the study. The complete description of the program flags can be found in the documentation of the respective programs.

### ****Cutadapt****

**Source:** <https://pypi.python.org/pypi/cutadapt/1.1>**;**

**Version: 1.1**

**Parameter settings:**

**cutadapt -a / -n options -O 10 -q 10 -m 25 –match-read-wildcards).**

### Sff_extract

**Source:** http://bioinf.comav.upv.es/sff_extract/;
**Version:** 0.3.0

****Parameter settings:****

sff_extract -c --min_left_clip=16 FGRYXA301_Region1.sff FGRYXA302_Region2.sff -o 454_gerald_clipped

### Reptile

**Source:** http://aluru-sun.ece.iastate.edu/doku.php?id=reptile;

**Version:** 1.1

****Parameter settings:****

QBVflag 1 , BatchSize 1000000, KmerLen 13, hd_max 1, Step 13, ExpectSearch 16, T_ratio 0.2, Qthreshold 94, MaxBadQPerKmer 3 , Qlb 80, T_expGoodCnt 80, T_card 12

### ****ABySS Illumina+454****

**Source:** <http://www.bcgsc.ca/platform/bioinfo/software/abyss>;
**Version:** 1.2.2

****Parameter settings:****

Additionally para meters: --erode=7, --erode-strand=1, --coverage=7.

### MIRA hybrid assembly

**Source:** <http://sourceforge.net/projects/mira-assembler/files/>;

**Version:** 3.2.1

****Parameter settings:****

mira -project=MIRA8 -fasta -job=denovo,est,accurate,sanger,454,solexa -notraceinfo -AS:sd=yes -AS:sd_last_pass_only=yes -CL:ascdc=no -CL:asjdc=no -CL:pec=no -GE:not=4 -CO:asir=no -LR:ssiqf=no -AS:sep=yes -AS:sdlpo=no -AS:ard=yes -AS:ugp=yes -OUTPUT:ora=yes -SK:pr=90
SANGER_SETTINGS -AL:mrs=98 -AL:min_overlap=35 -CO:rodirs=5 -CL:mbc=on -AL:egp=no -CL:qc=yes -CL:cpat=no
454_SETTINGS -AL:mrs=98 -AL:mo=35 -CO:rodirs=5 -ED:automatic_contig_editing=yes -AL:extra_gap_penalty=no -CL:cpat=no
SOLEXA_SETTINGS -AL:mrs=98 -AL:mo=35 -CO:rodirs=5 -AS:bdq=30 -AS:epoq=no -LR:ft=fasta -LR:wqf=off -CL:qc=no -CL:cpat=no

### RBR

**Source:** <http://malde.org/~ketil/biohaskell/rbr/>;

**Version:**  0.8.6

****Parameter settings:****

For Library-less repeat masking, RBR source code was downloaded the link above and compiled locally. The parameters used are:

rbr -s 1 -t 4 --preserve-lower-case -v -L +RTS -M20G -K32M -RTS

### TGICL

**Source:** http://compbio.dfci.harvard.edu/tgi/software/

**Version:** 2.1

****Parameter settings:****

TGICL (v2.1) was used with following parameters, tgicl -F <input.fasta> -c 15 -O '-p 97 -l 60'

### SnoWhite

**Source:** http://EvoPipes.net

**Version:** 1.1.3

****Parameter settings:****

perl snowhite_1.1.3.pl -f <*.fasta> -a B -t B -p 4 -b 10 -r 10 -m 1 -o <*.fasta>

### **Blastall**

**Source:** ftp://ftp.ncbi.nlm.nih.gov/blast/executables/blast+/2.2.28/

**Version:** 2.2.28

****Parameter settings** (Ribosomal RNA databases)**:****

blastall -p blastn -a 7 -d <LSURef_SILVA.fasta> -i <*.fasta> -m 7 -e 1e-10 -V T -I T > <LSU_BLAST.xml>

blastall -p blastn -a 7 -d <SSURef_SILVA.nr.fasta> -i <*.fasta> -m 7 -e 1e-10 -V T -I T > <SSU_BLAST.xml>

SwissProt

blastall -p blastx -a 15 -d <swissprot> -i <*.fasta> -m 7 -e 1e-6 -V T -I T > <SWISSPROT_BLAST.xml>

NCBI nr

blastall -p blastx -a 15 -d <nr> -i <*.fasta> -m 7 -e 1e-6 -V T -I T > NR_BLAST.xml

****Parameter settings** (*Hypsibius dujardini* ESTs)**:****

blastall -p tblastx -d <h_dujardini_EST.fasta> -i <*.fasta> -f 999 -a 15 -e 1e-6 -m 7 -V T -I T > h_dujardini_BLAST.xml
